# Sustainable circular biorefinery approach for novel building blocks and bioenergy production from algae using microbial fuel cell

**DOI:** 10.1080/21655979.2023.2236842

**Published:** 2023-07-23

**Authors:** Kevin Tian Xiang Tong, Inn Shi Tan, Henry Chee Yew Foo, Pau Loke Show, Man Kee Lam, Mee Kee Wong

**Affiliations:** a Department of Chemical and Energy Engineering, Faculty of Engineering and Science, Curtin University Malaysia, Miri, Sarawak, Malaysia; b Department of Chemical Engineering, Khalifa University, Abu Dhabi, United Arab Emirates; c Zhejiang Provincial Key Laboratory for Subtropical Water Environment and Marine Biological Resources Protection, Wenzhou University, Wenzhou, China; d Department of Chemical and Environmental Engineering, Faculty of Science and Engineering, University of Nottingham Malaysia, Semenyih, Malaysia; e Department of Sustainable Engineering, Saveetha School of Engineering, SIMATS, Chennai, India; f Chemical Engineering Department, Universiti Teknologi PETRONAS, Seri Iskandar, Perak, Malaysia; g HICoE-Centre for Biofuel and Biochemical Research, Institute of Self-Sustainable Building, Universiti Teknologi PETRONAS, Seri Iskandar, Perak, Malaysia; h PETRONAS Research Sdn Bhd, Kajang, Selangor, Malaysia

**Keywords:** L-lactic acid, microfluidic, electrofermentation, bioelectricity, 3D printing

## Abstract

The imminent need for transition to a circular biorefinery using microbial fuel cells (MFC), based on the valorization of renewable resources, will ameliorate the carbon footprint induced by industrialization. MFC catalyzed by bioelectrochemical process drew significant attention initially for its exceptional potential for integrated production of biochemicals and bioenergy. Nonetheless, the associated costly bioproduct production and slow microbial kinetics have constrained its commercialization. This review encompasses the potential and development of macroalgal biomass as a substrate in the MFC system for L-lactic acid (L-LA) and bioelectricity generation. Besides, an insight into the state-of-the-art technological advancement in the MFC system is also deliberated in detail. Investigations in recent years have shown that MFC developed with different anolyte enhances power density from several µW/m^2^ up to 8160 mW/m^2^. Further, this review provides a plausible picture of macroalgal-based L-LA and bioelectricity circular biorefinery in the MFC system for future research directions.

## Graphical Abstract

**Figure:**
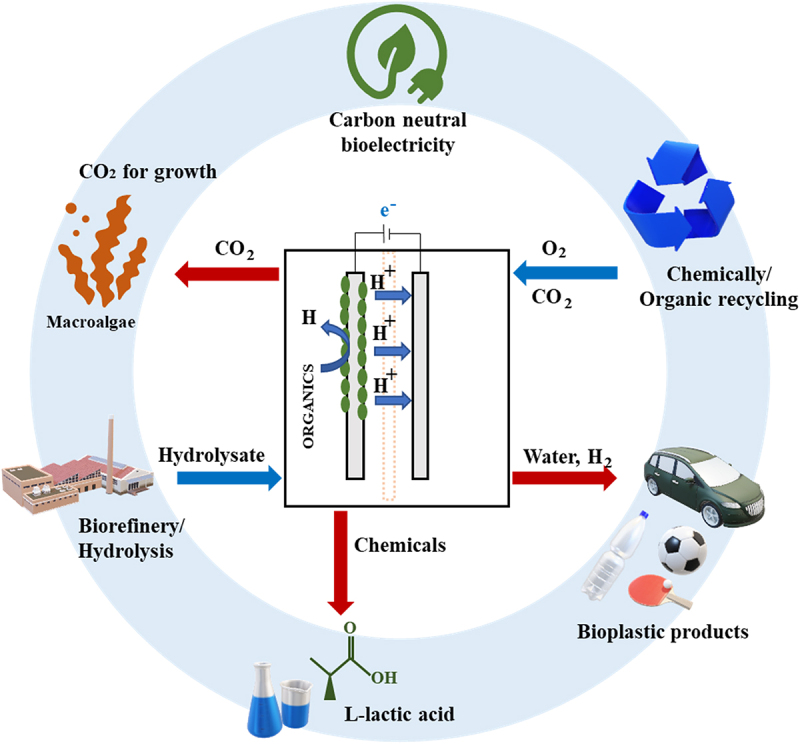

## 1. Introduction

Industrialization, the engine of urbanization and economic expansion, has accelerated the development of petrolic energy and polymer sectors in association with the growth of the global population and affluence [1]. Meanwhile, due to the outbreak of the COVID-19 pandemic, a nearly 130-fold increment of plastic waste has been perceived over the past 10 years (2011–2020) and reached approximately 8.51 billion tons as an unprecedented increase in single-use plastics (SUPs), including protective medical suits and face masks [2]. It is assessed that mismanaged plastic wastes are omnipresent and outweigh fish in the marine environment, with at least 7 million tons of plastic debris dumped into the ocean annually, which has caused catastrophic damage and deaths of marine animals [3]. Such vivacious SUPs production results in well-established concerns that are deleterious to the environment, such as land pollution, air pollution, and marine pollution that causes anthropogenic CO_2_ emissions. The International Energy Agency (IEA) website shows that the rise in carbon footprint from 2005 to 2021 has reached an 18% increment [1]. This linear economy has been heavily reliant on the overexploitation of fossil fuels and natural resources, which drastically impairs the life of future generations [3]. Suppose the carbon footprint continuously rises at its current rate into the atmosphere. In that case, the dynamic equilibrium of the carbon cycle will diverge, resulting in irreversible changes in the climate system. Hence, sustainable practices and concerted efforts to minimize carbon footprint whilst simultaneously maintaining global economic growth have to be initiated through various technological interventions to meliorate the current linear economy toward a biobased bioeconomy.

In pursuing the United Nations’ 7^th^ Sustainable Development Goal (SDG), the bioeconomy has to be designed to generate affordable, clean, and sustainable energy [4]. From the point of view of sustainability, this concept covers three major points: (1) maximum utilization of organic resources; (2) minimum energy suffices the production processes; (3) the waste produced from one operation becomes the feedstock for another [5]. Notably, the circular bioeconomy model has drawn significant attention to address the environmental burden and resource depletion caused by the linear economy model. In the circular bioeconomy model, the maximum energy potential and value of the biomass can be aroused, extracted, and retained to their maximum extent via the circular biorefinery approach. The circular biorefinery approach is a regenerative closed-loop method that conserves the longevous biomass resources to maximize economic productivity and minimize waste generation at the end of each service life, which helps mitigate the pressure on the environment [6]. Recently, the concept of holistic zero-waste circular biorefinery has been successfully applied to the co-production of bioethanol and biogas (biomethane) from red macroalgae *Eucheuma denticulatum* residues by Loh et al. [7]. They concluded that the developed macroalgae-based circular biorefinery system demonstrated an exergy efficiency of 73.74% with a functional exergy efficiency of 15.30% and the cascading approach allowing an almost complete conversion of the macroalgal biomass [7]. A macroalgae-based circular biorefinery system for bioethanol and L-lactic acid (L-LA) was also established by Wong et al. [8] using red macroalgae *Eucheuma cottonii* residues, manifesting the electricity generated from the combined heat and power plant of the system proficient at supplying up to 70% of the plant’s total electricity requirement. Further, Grasa et al. [9] investigated the economic evaluation using brown macroalgae *Laminaria* sp. as biomass for large-scale L-LA production. They revealed that the minimum viable selling price is reduced by 34% compared with the current selling price (USD 3.83/kg), demonstrating an economic feasibility and sustainability biorefinery approach [9]. To this extent, the macroalgae biomasses tally with the circular biorefinery concept compared to lignocellulosic biomass (LCB), a cornerstone of the biorefinery to date due to non-competitiveness with food supply and fast growth rate [10,11].

To achieve carbon neutrality and a sustainable circular biorefinery, durable biobased products need to be developed without sacrificing performance. Various strategic paths to produce carbon-neutral macroalgal bioenergy and bioplastics have been mapped out with expanding research outputs [12,13]. At the upshot of these efforts, one of the latterly proposed alternative bioenergy sources is microbial fuel cells (MFC) which generate renewable electricity using active microorganisms or bacteria as a biocatalyst in an anaerobic anode chamber [13]. Gebreslassie et al. [13] revealed that brown macroalgae *Saccharina japonica* was successfully metabolized by methanogenesis in a H-shape dual-chamber MFC for bioelectricity production with a maximum power density of 1820 mW/m^2^. Further, Okoroafor et al. [14] detailly calculated the substitution potential of petrolic electricity with bioelectricity produced via MFC and evaluated global warming potential (GWP) impacts. Initially, the GWP was evaluated based on the United Kingdom’s electricity demand; further, GWP for bioelectricity MFC was evaluated for petrol-based electricity. It has been deduced from the calculations that 15.7–19.5 ktons of CO_2_ equivalents can be diminished by bioelectricity production of 10,030 GJ each year [14]. Furthermore, using renewable carbon in bioelectricity helps curtail the dependency on fossil fuels, which can further control or reduce the carbon footprint throughout this life cycle [15].

In addition to tremendous efforts to reverse global climate change, a valuable novel building block for poly-L-lactic acid (PLLA) bioplastic, notable L-LA, can also produce from macroalgal biomass via the biotechnological approach by using lactic acid bacteria (LAB) to valorize carbohydrate contents [11]. PLLA is a biodegradable thermoplastic with properties similar to petrolic plastics (polypropylene, polyethylene, polystyrene, and polyamide) and offers added advantages due to generating a lesser carbon footprint on the environment with a feasible end-of-life strategy [16]. By the biorefinery principle Task 42, the co-production of bioenergy and value-added biochemicals from biomass in an integrated system are advocated to advance the material wealth of human society in terms of materials and energy supply for a new sustainable industrial development [17]. In an evaluation of the carbon footprint of biorefinery producing biochemicals and bioenergy from wastewater in an MFC model using an attributional life cycle assessment (LCA) assay, the authors revealed that the best scenario was the integration of the production chain compared to standalone production, resulting in a 112.18% diminution to the net system emission by attaining −2.2 global warming ratio compared to standalone processing [18]. Thus, a cascading macroalgal biorefinery system in the MFC model is recommended for a zero-waste conversion technology to address the dilemma of bioelectricity and L-LA. Once fully optimized, the cascading macroalgal biorefinery system has the potential to transform the energy and polymer sector into a climate-neutral hub whilst helping the circular bioeconomy.

This paper is systematically designed to critically review the successful development of bioelectricity and L-LA from macroalgal biomass for bioenergy and bioplastic applications. The prospects and state-of-the-art technological development of MFC for integrated bioprocesses as a strategy for carbon neutrality and sustainability were enclosed. In addition, the advantages of key bioprocesses that are employed for macroalgae valorization for L-LA and bioelectricity production were also discussed extensively. Based on the different technological employment, some recommendations were made for future research directions on the seamless integration of macroalgal-based L-LA and bioelectricity production. Hence, this review provides essential technical enlightenment on the contemporary status and future trends of MFC applications in pursuit of a sustainable circular biorefinery.

## 2. Marine macroalgae: the future of sustainable bioenergy and bioplastic

**Figure 1.**
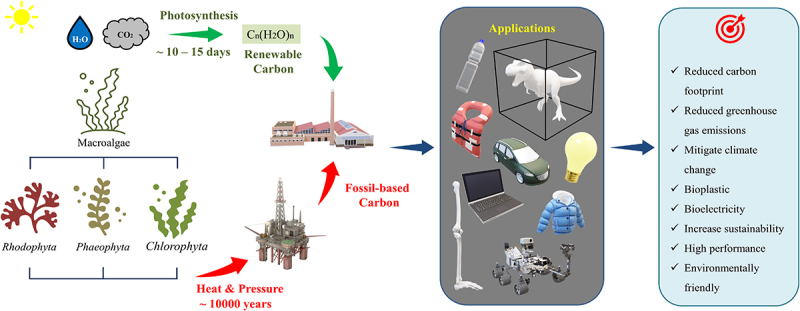
Schematic representation of macroalgal bioelectricity and bioplastics from cradle-to-gate perspectives for durable applications.

The research interest in utilizing marine and cultivated macroalgal biomass began to arise once hindrances associated with LCB emerged, mainly the harsh thermochemical pretreatment involved in removing the lignin complexes [19]. Among 29,800 identified macroalgae strains, merely 230 strains have been studied for biorefinery, and their potential for miscellaneous applications is irrefutable [20]. The cradle-to-gate delineation of the development of macroalgal bioelectricity and bioplastics is illustrated in Figure 1. Macroalgae are aquatic photosynthesis and carbohydrate-rich multicellular eukaryotic organisms ubiquitously present in marine habitats, hence the acronym ‘marine plants’ or ‘seaweeds.’ Macroalgae are of three types based on the natural pigmentation type: red (*Rhodophyta*) taxonomical group colored by phycoerythrin, brown (*Phaeophyta*) taxonomical group colored by fucoxanthin, and finally green (*Chlorophyta*) taxonomical group colored by chlorophylls. All taxonomical groups contain specific cell wall polysaccharides such as cellulose and phycocolloids in varying compositions, which contribute to the diversity of macroalgal species. Floridean starch, laminarin and amylopectin are usually the storage polysaccharides for red, brown, and green macroalgae, respectively. Their lack of recalcitrant lignin complexes indicates that less energy-intensive bioprocesses can be employed to recover value-added bioproducts of commercial interest, favoring techno-economic and LCA analyses of any potential biorefinery processes that utilize macroalgae as feedstock [8,10]. Moreover, high and specialty easy-degradable polysaccharides (65–95 wt%) in each taxonomical group of macroalgae species offer extensive features for either platform compounds or direct use in the biorefinery [21].

**Figure 2.**
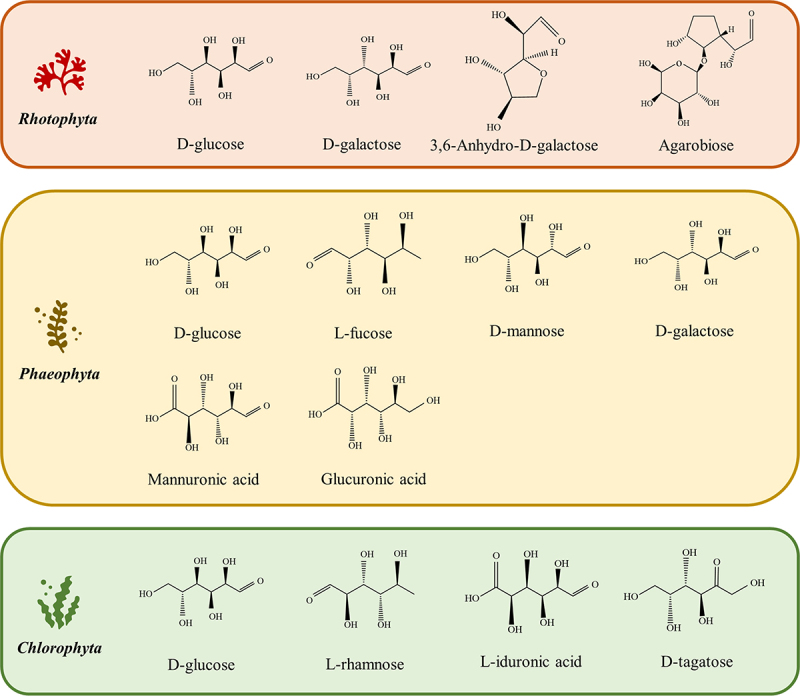
Major monosaccharides present in the hydrolysates of red, brown, and green macroalgae.

From the perspective of bioelectricity generation and L-LA fermentation, the significant disparity between the three macroalgae species relies upon their inherent sugar composition, and the conversion yield will be evaluated by the fermentation performance of the endowing lactic acid bacteria on the derived rare sugars. Figure 2 depicts the major macroalgae-derived rare sugars via hydrolysis, revealing that rare sugars in the form of D-glucose can be found in all macroalgae taxonomical groups representing the appearance of cellulose compounds in the macroalgal cell wall [11]. Red macroalgae, with *Gracilaria* sp. and *Eucheuma* sp. as the representative species, possess agarose and carrageenan as the heterogeneous phycocolloids composed of agarobiose, D-galactose, and 3,6-anhydro-D-galactose, of which 3,6-anhydro-D-galactose is non-metabolize [12,22]. Brown macroalgae are copiously cultivated for the extraction of alginate, laminarin, mannitol, and fucoidan, which is widespread in food processing applications. L-fucose, D-mannose, D-galactose, mannuronic acid, and glucuronic acid are the major monosaccharides in brown macroalgae hydrolyzates [23,24]. In addition, green macroalgae mainly embrace ulvan as the sole heterogenous polysaccharide composed of L-iduronic acids and rare sugars, including L-rhamnose and D-tagatose [25]. Thus, macroalgae are considered sustainable sources of rare sugars for biorefinery purposes. It also addresses the sustainability concerns related to food security and arable land suffered by the edible crops and LCBs, as macroalgae are abundant in supply and generally grow in marine habitats [11].

Furthermore, macroalgae are considered a year-round available feedstock for biorefinery purposes due to their bioavailability properties with a short average growth period of 10–15 days [26]. The global industry exploits 35.08 million tons of 230 macroalgae species cultivated worldwide in 2020, dominated by red macroalgae species, which seizes 18.84 million tons and corresponds to 53.70% of the total productivity [27]. Besides, the global annual production of wild stock macroalgae in seashore without any farming techniques bestows a net primary production (NPP) of 1.32 Pg carbon per year, where this productivity is aligned with the Amazon rainforests. NPP is defined as the balance between photosynthetic CO_2_ sequestration and CO_2_ released by autotrophic respiration, in which the positive NPP value describes the environment possessing high initial biomass and higher biomass productivity [28]. Alongside macroalgae propagation from aquaculture or wild stocks as the feedstock for biorefinery purposes, carbon offsetting can occur simultaneously by photosynthetic CO_2_ sequestration. This is mainly due to macroalgal habitats being the most extensive and productive of all coastal vegetated ecosystems. Raven [29] revealed that strongly autotrophic macroalgae communities globally sequester approximately 1.6 gigatons of carbon/year through their net production. Regarding climate change, Seghetta et al. [30] concluded that the net removal of CO_2_ from the atmosphere by macroalgae aquaculture and biorefinery is about 190 kg of CO_2_ equivalents per hectare of cultivation area. From the perspective of CO_2_ sequestration, the intense photosynthetic activity by macroalgal forests obviates ocean acidification, resulting in a balanced pH for marine ecosystems. In fact, ocean acidification represents a threat that negatively impacts the growth and survival of calcifying organisms, including coral reefs, oysters, shrimps, and clams by wresting the minerals used by these organisms to build their shells and skeletons [31].

In addition, bioenergy and bioplastics produced from macroalgal biorefinery will be zero net addition of CO_2_ to the atmosphere; any CO_2_ emitted in combustion originated from that sequestered during recent photosynthesis and can potentially be recaptured in the same way [1]. Moreover, Plastics Europe Association (PEA) conducted the LCA of macroalgal-based bioplastic and concluded that macroalgae biomass has substantial potential to produce value-added biochemicals such as L-LA for bioplastic production [32]. In short, the round-of-the-year availability of macroalgal biomass, the ability for CO_2_ sequestration, and high carbohydrate content increase their potential for bioenergy and bioplastics production.

### 2.1. Bioenergy

**Figure 3.**
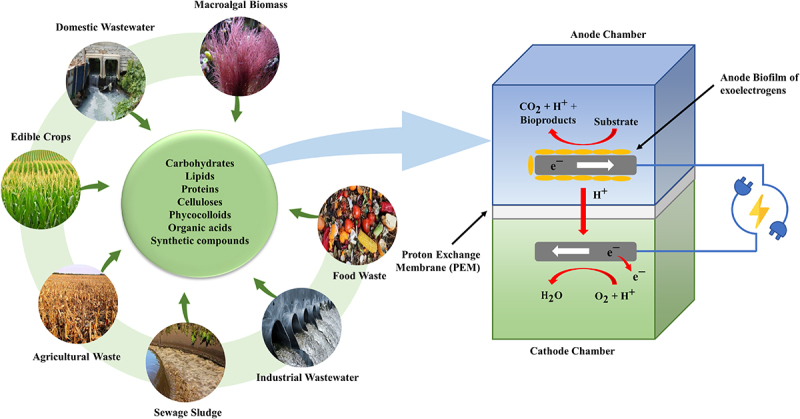
Microbial fuel cells (MFC) for bioelectricity production from a variety of biomass and waste sources.

Interest in biomass-to-energy (B2E) strategies has peaked in the quest for establishing a carbon neutral cycle by drawdown CO_2_ released, solving both energy and environmental crises simultaneously. Renowned for its reliable and developed technology, B2E is an eco-friendly approach that can generate green energy from biomass or post-consumer waste [33,34]. Among the available B2E technologies, MFC has demonstrated promising prospects in directly converting biomass to bioelectricity over exoelectrogens (anodophilic) microorganisms [15]. In general, the bioconversion of organic matter for bioelectricity production from MFC could be depicted in Figure 3. The MFC consists of two chambers, an anode chamber and a cathode chamber, separated by a proton exchange membrane (PEM). Further, the exoelectrogens are typically immobilized on the surface of the anode, resulting in a biofilm over the anode surface to debase the biomass, releasing electrons and protons, which later be used for bioelectricity production [35]. The bioelectricity potential and vitality transformation of MFCs rely significantly on the efficiency level attained by both the biodegradation process and electron transfer [36]. Compared to conventional anaerobic degradation, MFC manifests greater potential with its higher energy recovery efficiency from biomass [13]. Typically, conventional anaerobic degradation produces biogas from biomass, which is susceptible to 20% entropy lost during its combustive conversion into useable bioelectricity [8]. Contrarily, such combustive conversion is negligible for MFC as its mechanism involves coupling exoelectrogen metabolism and electrochemical reaction to convert the chemical energy present in the biomass into usable electrical energy. Moreover, MFC can also operate under ambient temperature, thus offering a higher safety index and exergy efficiency for bioelectricity generation [37].

Gebreslassie et al. [13] proved the feasibility of macroalgae for the co-production of bioelectricity and hydrogen by applying red macroalgae *Saccharina japonica* as substrate in MFC. The authors revealed that energy recovery of 17.3% was attained with an optimum power density and hydrogen production of 1.82 W/m^2^ and 110 mL/g, respectively [13]. As a further illustration, *S. japonica* substrate in the anode chamber is oxidized by fermentative bacteria under dark fermentation operation to produce hydrogen; however, carboxylic acids, including acetic acid, butyric acid, propionic acid, and lactic acid (LA) were produced simultaneously as the by-products. The produced carboxylic acids are strongly hydrophilic; thereby, the recovery of carboxylic acids from fermentation broths requires a complicated separation process, which hinders their practical application [13]. Hence, the produced carboxylic acid molecules [composition: R-COOH] participated in the anodic reaction and oxidized by exoelectrogens into a reduced form for electrons and protons production, which can be depicted in Eq. (1). The released electrons from the anodic reaction are then transferred to the anode through the exoelectrogen cell membrane, where anode acts as a terminal for the electron transfer process to the cathode chamber. At the cathode chamber, the electrons transported from the anode combine with the protons, which traverse the PEM, resulting in the creation of water molecules in the presence of oxygen [38]. This reaction completes the electron flow in the circuit, balancing the charges caused by the anodic reaction and generating electric current.

Notably, CO_2_ is released as a biogenic by-product of microbial respiration and decomposition of biomass during the anodic reaction, in which the exoelectrogens and fermentative bacteria in the anode chamber of the MFC oxidize *S. japonica* substrate as their energy source. From Eq. (1), R-COOH compounds are broken down, releasing biogenic CO_2_ as an inherent metabolic by-product, considered a natural carbon cycle [13]. Interestingly, the released biogenic CO_2_ can indirectly help mediate the pH shift between the two chambers by participating in the carbonate buffering reactions, shown in Eq. (3) [35]. Okoroafor et al. [14] concluded that adding buffer salts such as phosphates and carbonates to the anolyte and catholyte is not economically feasible for large-scale production and will contribute to an 80% increment of GWP compared to CO_2_ as a buffering agent. Furthermore, an integrated MFC system for bioelectricity and carbon capture was developed by Zhang et al. [39] using an algal biocathode with a layer of biofilm consisting of photosynthetic algae *Chlorella vulgaris*. With this technique, the released biogenic CO_2_ will be supplied to promote better growth and photosynthesis of the algae biomass [composition: (CH_2_O)_n_], improving carbon fixation [39]. Eq. (4) depicts that oxygen is generated as an electron acceptor by photosynthesis, which later is used in the cathodic reaction, creating a sustainable cycle loop. Thus, MFC aligns with green chemistry and provides a framework for contributing to achieve the carbon neutrality goal as they operate under mild conditions to convert the chemical energy of biomass to electrical energy through microbial metabolism. The detailed reaction for carbon capture and fixation is further discussed in Section 3.2.

**Table:** 

Anodic reaction: R-COOH + H_2_O $\;\vec{Exoelectrogens}$ 2CO_2_ + 8H^+^ + 8e^−^	(1)
Cathodic reaction: 8H^+^ + 8e^−^ + 2O_2_ → 4H_2_O	(2)
Carbonate buffering reaction: CO_2_ + H_2_O ↔ H_2_CO_3_ ↔ H^+^ + HCO_3_^−^	(3)
Photosynthesis: nCO_2_ + nH_2_O → (CH_2_O)_n_ + nO_2_ (in the presence of algae)	(4)

### 2.2. Bioplastic

**Figure 4.**
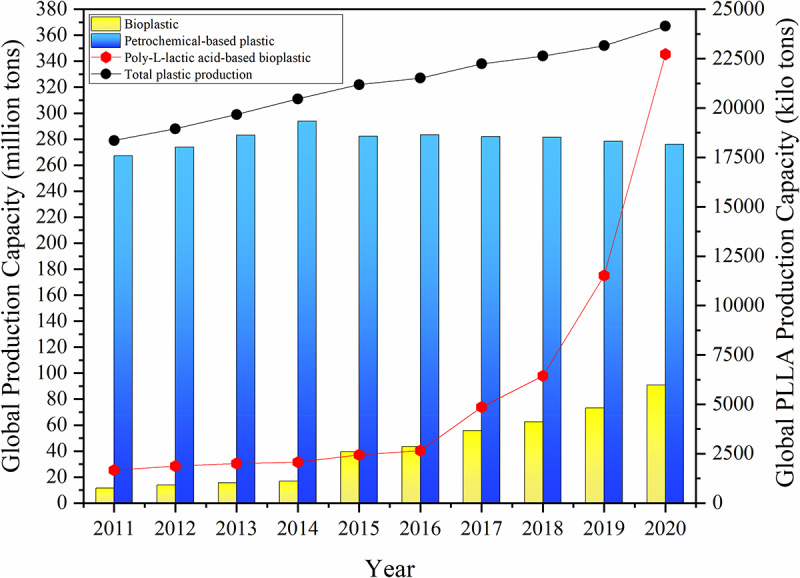
World production of synthetic plastics and bioplastics from 2011 to 2020. Adjusted from [40].

Over the years, petrolic plastic consumption and production have grown significantly, owing to its adaptability and affordability, making it ideal for widespread applications [3]. Global plastics production has reached over 360 million tons annually, dominated by petrolic plastics with seizes 276 million tons and up to 50% of that is for single-use purposes (Figure 4) [40]. Jem and Tan [41] reported that 82% of plastic waste from total plastic production was excluded from recycling, in which the majority of plastic waste was either heaped in landfills or cremated, with the remainder spewed into the ocean as litter. As for the plastic waste dumped into the oceans, the carbonyl group of petrolic plastic absorbs UV radiation below 280 nm, making the polymer susceptible to photo-oxidative degradation, breaking down into petrolic microplastics [40]. Petrolic microplastic contamination has reached an alarming level as they are ubiquitous and available for ingestion by aquatic organisms. These aquatic organisms will suffer from entanglement and starvation as their stomachs become filled with plastic, leading to death. Moreover, petrolic microplastics can cause genotoxicity and immunotoxicity to marine life as they contain polycyclic aromatic hydrocarbons [42]. Recent research has shown overwhelming evidence that microplastics are in tandem with other toxic chemicals (heavy metals and organic metals), serving as a vector for their transport in the environment, which then be enriched in the food chain to endanger not only marine life but also human beings [43]. Besides, the accumulation of plastic waste in landfills is a growing environmental concern as petrolic plastics take around a century or more to degrade. During thermal-oxidation degradation of petrolic plastics, methane and ethylene will be produced and contribute to CO_2_ emission when exposed to sunlight and heat, impacting human health and climate [3].

The awareness of the pressing environmental issue regarding plastic pollution has diverted attention toward the utilization of alternatives, such as biodegradable plastic (bioplastic) derived from a biopolymer. Unlike petrolic plastics for remaining unchanged for centuries, bioplastics can be degraded relatively quickly by the action of microorganisms, namely biodegradation, with a specific surface degradation rate of 21 µm/year under specific conditions [44]. Bioplastics can be broken down into nutrient-rich biomass, CO2, and inorganic compounds, leaving no toxins and residue behind. The nutrient-rich biomass can be converted into fertilizer or utilized as feedstock for biorefinery purposes, while the CO_2_ released can be sequestrated for photosynthetic activity by Plantae [45]. Thus, CO_2_ emissions are avoided as no carbon is involved in the manufacturing process of bioplastics. One of the most extensively researched biopolymers for bioplastics is polylactic acid (PLA). PLA is a biodegradable aliphatic thermoplastic polyester and has excellent market prospects, with a range of applications from industrial to civilian use [46]. Briefly, PLA can be defined as a chain-formed oligomer of LA that polymerizes by either a Ring-Opening reaction of lactide or by a Direct Poly-Condensation of high-purity LA that can be derived from macroalgal biorefinery [41,47,48].

Its monomer, LA, can be found in either D(-) or L(+) enantiomeric forms due to an asymmetric carbon atom, but L-LA is the dominant isomer for commercialized PLA. Compared to L-LA, D-LA usage in biomedical applications is skeptical due to its harmful to human health, especially will cause neurotoxicity in the human body [49]. This is because L-LA is the primary endogenous agonist of hydroxycarboxylic acid receptor 1 (HCA_1_), which naturally occurs in living organisms and the human body has enzymes and metabolic pathways specifically designed to metabolize L-LA. Contrarily, D-LA is not naturally produced by the human body and the enzymes do not efficiently metabolize D-LA, leading to the accumulation of D-LA in the body, which can disrupt the normal physiological processes of human beings [50]. Excessive accumulation of D-LA can result in acidosis, an abnormal decrease in blood pH, affecting detrimental effects on various bodily functions. Prolonged exposure to acidic conditions can be detrimental to cells and tissues. Further, heaping D-LA can induce inflammation and impede tissue healing and generation, making D-LA not suitable for biomedical applications [49]. Hence, good biocompatibility is an important reason for the wide use of poly-L-lactic acid (PLLA) in the field of biomedicine.

Globally, a nearly 14-fold increment of PLLA has been produced compared to 2011, reaching circa 22.72 million tons in 2020 [40]. The drastic growth in annual PLLA capacity (25% of bioplastic production, Figure 4) stems from its high mechanical strength and durability properties similar to petrolic plastics [46]. The mechanical performance of PLLA is characteristic of glassy polymers with low deformation and high melting temperature (160–180°C), which is suitable for different processing means, including melt-extrusion molding, vacuum molding, foam molding, and injection molding. The good flexural strength (up to 140 MPa) and processing ability of PLLA yield various plastic products, such as bioplastics, filament for fused deposition modeling, and fabrics for industrial and civilian use [46,51]. With its biocompatibility properties and approval by the United State Food and Drug Administration (FDA) for biomedical applications, high molecular weight PLLA has been used to produce non-dismantling surgical sutures and bone screws, while low molecular weight PLLA as a slow-release drug-packaging agent [45]. Casalini et al. [52] manifested that the degradation of PLLA material by the enzymes produced by the human body can be used for drug release. By controlling the metabolism rate and composition of PLLA material in the capsules, the therapeutic drugs can quickly target the lesion site, improve its bioavailability, and reduce its toxic side effects and toxicity to other tissue types, leading to a benefit for drug delivery and as a material for implants structure [52].

To comprehensively compare PLLA with petrolic plastics from the point of view of carbon footprint, a cradle-to-grave LCA along with Intergovernmental Panel on Climate Change method was performed by Morão and de Bie [53]. The authors revealed that the production cycle of PLA-based plastic demonstrated lower environmental burdens than petrolic plastic by uptaking 1.8 tons of CO_2_ equivalents per ton of plastic produced [53]. In addition, Abdul-Latif et al. [54] revealed that the annual production of macroalgal-based PLLA should be more than 600 million tons to replace the petrolic plastics for achieving carbon neutrality, which is calculated by using carbon emission pinch analysis with total CO_2_ emission levels and plastic production in the view of the year 2016 as base study. Based on the current PLLA production data, there is an urgent need to accelerate our efforts to increase macroalgae-based L-LA production to meet the high demand for PLLA. Besides diminishing carbon footprint, Coppola et al. [55] also reported that the production of PLLA saves circa two-thirds of the energy required for petrolic plastics production, which offers an energy-effective process. Thus, macroalgal-based PLLA is a promising solution to defossilize the economy.

In order to meet the global market demand, large amounts of macroalgal-based L-LA are essential for the industrial-scale production of PLLA. L-LA can be derived by chemo-catalytic or microbial fermentation processes. The chemo-catalytic process uses petrolic chemicals such as propionaldehyde and acetaldehyde as feedstock which may be subject to the potential supply problem of crude oil and its dramatic price variation. The drawback of the chemo-catalytic process is that it involves environmentally unfavorable chemicals (hydrogen cyanide, sulfuric acid, and sodium hydroxide) and produces only the racemic (50:50) mixture of L-LA and D-LA, which is not desirable for food and biomedical applications due to the metabolic issues that D-LA may cause. Further, the racemic mixture is not feasible for PLA production, typically requiring LA with high optical purity (e.g. ~99% L-LA or ~ 99% D-LA) [56]. Then, the microbial fermentation process outcompetes the chemo-catalytic process for high optical purity L-LA production as particular microbes have a specificity of *ldhL* gene that secretes L-lactate dehydrogenase (L-LDH) enzymes, which can only convert pyruvate to L-LA. Typical microbes employed for L-LA production are low guanine and cytosine-content Gram-positive bacteria, namely, lactic acid bacteria (LAB) [57]. LAB is a group of microorganisms that belong to the family Lactobacillaceae and is characterized by their ability to produce L-LA via carbohydrate metabolism. Being considered the macroalgal hydrolyzate composed of mixed rare sugars, which need to be completely metabolized for better L-LA conversion yield, the LAB offers an excellent regulatory mechanism known as carbon catabolite repression (CCR) for efficient carbohydrate metabolism [58].

CCR is the central governing mechanism in LAB for the regulation of rare sugar uptake, which ensures cellular resource efficiency by selectively regulating the uptake and metabolism of favorable carbon sources in the presence of non-favorable carbon sources, leading to both the favorable and non-favorable carbon sources can be metabolized albeit in a different rate [59]. In brief, CCR in LAB is regulated by different elements participating in rare sugar transport and metabolism, including sugar phosphotransferase system (PTS), enzyme I (EI), phosphocarrier histidine protein (HPr), and catabolite control protein A (CcpA) acts as the *trans*-acting repressor [60]. PTS is a complex transport system responsible for the uptake and phosphorylation of various rare sugars in the macroalgal hydrolyzate to acquire energy and regulate carbohydrate metabolism. The PTS transporters are sugar specific and phosphorylate the incoming sugar at the expense of phosphoenolpyruvate (PEP), mirroring the most suitable carbon sources available in the hydrolyzate. Notably, the energy for sugar transport is derived from PEP by donating the phosphoryl group as an energy carrier through the phosphotransferase reaction. This reaction converts PEP to pyruvate and transfers the phosphoryl group to a histidine residue on EI. EI, in turn, transfers the phosphoryl group to HPr. From the HPr, the phosphoryl group is transferred to the permease complex of the LAB cell. Then, the phosphoryl group is further transferred from the permease complex to the incoming sugar molecules, resulting in sugar phosphorylation. This phosphorylation step enhances the diffusion of sugar into the LAB cell and contributes to its subsequent metabolism [61]. Further, CcpA is a regulatory protein that acts as a transcriptional regulator facilitated in mediating CCR by controlling the expression of genes involved in metabolizing non-favorable carbon sources when the favorable carbon source is available. The CcpA will bind to specific DNA sequences called catabolite-responsive elements (*cre*), which are generally found in the promotor regions of target genes. The binding of CcpA to *cre* sequences modulates gene expression, leading to activation or repression of target genes involved in the utilization of non-favorable carbon sources, which further enhances carbohydrate metabolism [60]. Along with this functionality, Ahorsu et al. [62] revealed that the probiotic-based LAB *Bacillus coagulans* can simultaneously metabolize xylose in the presence of glucose for L-LA production. Besides, mesophilic LAB *Lactobacillus plantarum* also showed clear diauxic growth in a glucose-mannose-galactose mixture by attaining a L-LA conversion efficiency of approximately 65% [47].

**Figure 5.**
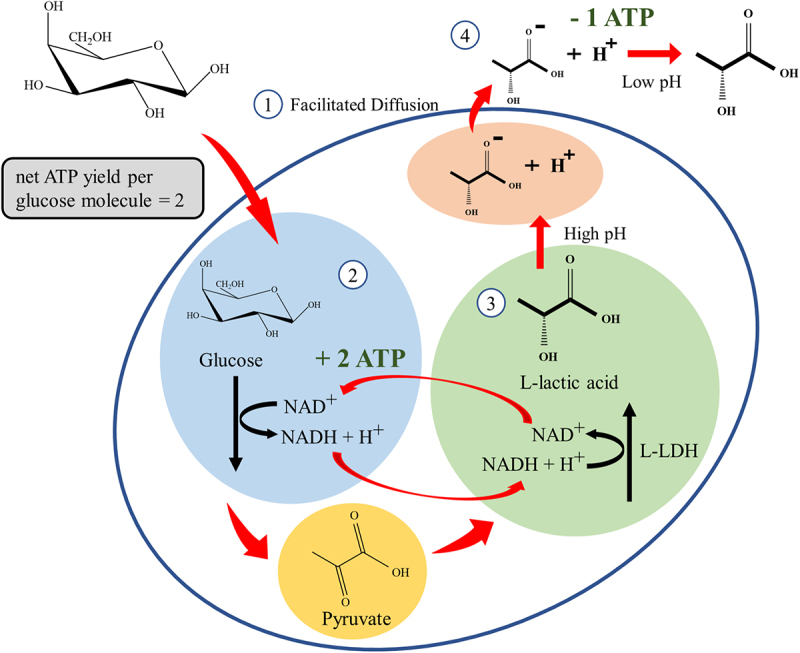
Simplified illustration of the cellular mechanisms involved in lactic acid production under homolactic fermentation and self-inhibition in the cytoplasm of *Lactobacillus delbrueckii* subsp. *bulgaricus* in anaerobic wort fermentation.

**Figure 6.**
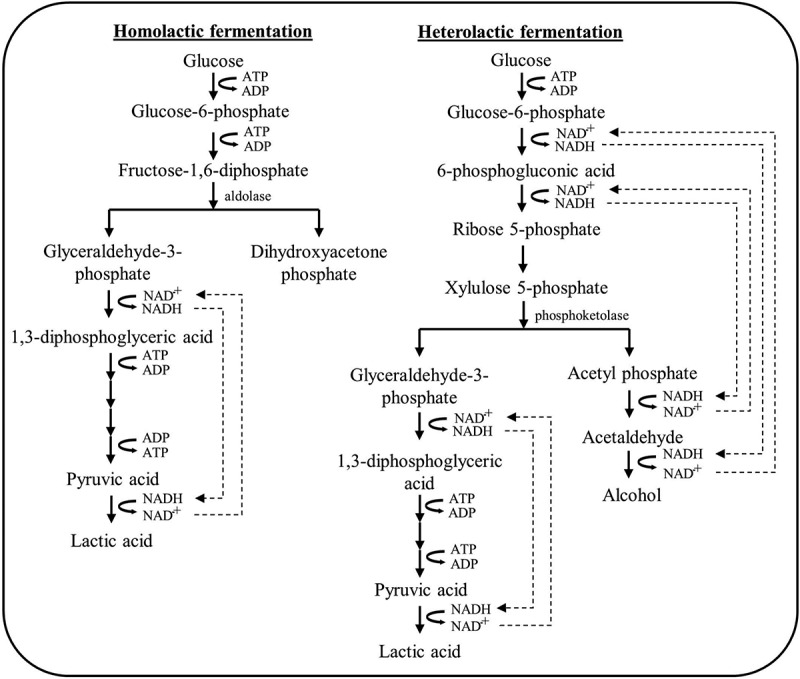
Mechanism of glucose glycolysis for lactic acid formation by lactic acid bacteria.

Depending on the particular LAB strain and the specificity of aldolase, the L-LA fermentation pathway can be divided into homolactic fermentation and heterolactic fermentation process. LAB possesses the aldolase enzyme is considered the homofermentative LAB, which can convert the carbohydrate-rich hydrolyzate exclusively into L-LA. Homofermentative LAB includes the strain from *Lactococcus*, *Streptococcus*, *Pediococcus*, *Lactobacillus*, and *Enterococcus* sp [57,63]. Due to the high energy yield characteristic that is beneficial for cell anabolism and cellular growth, the glucose molecule is the favorable carbon source for most of the LAB strains. In this perspective, glucose molecules will be metabolized via the Embden-Meyerhof glycolytic pathway by the homofermentative bacteria. For an insightful understanding of the Embden-Meyerhof glycolytic pathway, the glucose metabolism by homofermentative LAB, *Lactobacillus delbrueckii* subsp. *Bulgaricus* is illustrated in Figure 5. Glucose molecules are phosphorylated to glucose-6-phosphate (G6P) and transported into the cell by facilitated diffusion through PTS at the expense of one adenosine triphosphate (ATP) molecule. The ATP yield per glucose molecule diffused inside the cell is 2. Then, the G6P will convert to pyruvate by glycolysis yielding two molecules of ATP and one molecule of reduced nicotinamide adenine dinucleotide (NADH) per pyruvate [63]. The overview of the main steps and reactions involved in the glycolysis pathway is depicted in Figure 6. During the homolactic fermentation process, the G6P is phosphorylated by the enzyme phosphofructokinase-1, consuming one ATP molecule, forming fructose-1,6-diphosphate (FDP). The FDP is then cleaved into three-carbon molecules: dihydroxyacetone phosphate (DHAP) and glyceraldehyde-3-phosphate (G3P). DHAP is an isomer for G3P, but only G3P can directly continue through the next steps of glycolysis. Although both three-carbon molecules exist in equilibrium, but the equilibrium is ‘pulled’ strongly downward as G3P is depleted. Thereby, DHAP will convert into G3P by the enzyme triose phosphate isomerase, ensuring full utilization of both molecules. The G3P molecules undergo oxidation by the enzyme glyceraldehyde-3-phosphate dehydrogenase, resulting in the production of NADH and the formation of 1,3-diphosphoglycerate (1,3-DPG). NADH carries high-energy electrons that will later be used in ATP synthesis. Subsequently, 1,3-DPG donates one phosphate group to the adenosine diphosphate (ADP) molecule for producing an ATP molecule, forming 3-phosphoglycerate (3-PG). For further reaction, the 3-PG is converted into its isomer, 2-phosphoglycerate (2-PG). Then, 2-PG loses a molecule of water via a dehydration reaction catalyzed by enolase, becoming PEP. PEP is an unstable molecule, poised to transfer its phosphate group to ADP molecules for ATP generation. As it loses its phosphate, PEP is converted to pyruvate, the end product of glycolysis. This reaction is catalyzed by the enzyme phosphoglycerate kinase [64]. Consequently, L-LA is produced by the action of L-LDH on the pyruvate. On a molecular level, the metabolization of pyruvate by L-LDH is an additional means of NADH recycling. NADH plays a crucial role in the recycling of NAD^+^ to maintain glycolysis and the production of ATP through the L-LA fermentation, allowing the continuous breakdown of glucose and energy production under anaerobic conditions. The recycling pathway for NADH can be described as follows: (1) During glycolysis, the NAD^+^ is reduced to NADH when G3P is converted to 1,3-DPG; (2) During L-LA fermentation, a hydride ion (H^−^) from NADH is transferred to pyruvate for the formation of L-LA, resulting the regeneration of NAD^+^. By regenerating NAD^+^ from NADH, L-LDH ensures the availability of NAD^+^ for the continuation of glycolysis [65]. However, the produced L-LA has to be actively transported out of the cell at the expense of an ATP molecule. This is because of the fact that at high intracellular pH, L-LA dissociates into lactate and a proton. By maintaining the intracellular pH and proton motive force, this proton has to be exported via the plasma membrane proton ATPase at the expense of one ATP per proton. Once exported, at a low extracellular pH, the L-LA is again present in its protonated form. In theory, one molecule of glucose can produce two molecules of L-LA through homolactic fermentation [66].

In contrast to homofermentative LAB, heterofermentative LAB can decompose carbon sources into a variety of biochemicals, including L-LA, acetic acid, bioethanol, and CO_2_. Whereas CO_2_ gas detection is a diagnostic test for heterofermentative from homofermentative fermentation. Heterofermentative LAB includes the strain from *Leuconostoc*, *Oenococcus,* and *Weissella* sp [57,63]. The typical reaction pathways of heterofermentative fermentation include the phosphogluconate pathway, pentose phosphate pathway, and phosphoketolase pathway (Figure 6). During the phosphogluconate pathway, the glucose molecules are initially phosphorylated into G6P through the process of PTS and participate in the glycolysis process, which occurs in the cytoplasm of the LAB cell. The G6P is further oxidized by the enzyme glucose-6-phosphate dehydrogenase, resulting in the production of one molecule of NADH and 6-phosphogluconic acid. Through the pentose phosphate pathway, two half-reactions occur simultaneously: (1) 6-phosphogluconic acid is oxidatively decarboxylated by the enzyme 6-phosphogluconate dehydrogenase; (2) NAD^+^ is reduced to NADH. The overall reaction is exergonic, releasing energy that is then used to decarboxylate the 6-phosphogluconic acid, generating CO_2_ and ribulose 5-phosphate (Ru5P). The Ru5P molecule is then converted into other pentose phosphate sugar, ribose-5-phosphate (R5P) or xylulose-5-phosphate (X5P), which is catalyzed by the enzyme phosphopentose isomerase. The interconversion of Ru5P and R5P allows the synthesis of essential cellular components, including nucleotides and coenzymes for cell propagation. Subsequently, the X5P molecule is subjected to the phosphoketolase reaction and cleaved by the enzyme phosphoketolase, resulting in the production of two molecules including G3P and acetyl phosphate (AcP). Then, the G3P generated from the phosphoketolase reaction is further metabolized through the usual glycolytic pathway, similar to the steps in the conventional Embden-Meyerhof glycolysis pathway. Whereas G3P will enter the downstream steps of glycolysis, leading to the production of ATP and L-LA. While the other product of the phosphoketolase reaction, AcP is decarboxylated by the enzyme pyruvate decarboxylase, resulting in the release of CO_2_ and the formation of acetaldehyde. Lastly, acetaldehyde is reduced by the enzyme alcohol dehydrogenase, utilizing NADH as a cofactor. This step converts acetaldehyde into bioethanol [64]. In theory, one molecule of glucose can produce one molecule of L-LA and one molecule of bioethanol through heterolactic fermentation process [66]. However, the CO_2_ release and co-production of L-LA and other organic acids, which required additional separation and purification processes also hindered the employment of heterofermentative LAB for microbial fermentation [57]. Therefore, the utilization of homofermentative LAB is preferred over heterofermentative LAB for L-LA and PLLA commercial production due to it offering the high yield, productivity, and optical purity of L-LA.

## 3. Microbial fuel cell (MFC)

Contingent upon several cathodic reactions, MFC diversification has been developed to explore the particularity in the mechanism of distinct processes while enhancing the product output. Multi-faced applications based on the eventual anticipated product reorientated the nomenclature of MFC as microbial electrosynthesis system (MES), microbial desalination cell (MDC), electro-fermentation (EF), and microbial electrolysis cell (MEC). However, EF is explicitly applied for the production of a broad range of bioenergy and biochemicals, potentially achieving higher conversion efficiency (88%) than other MFC systems (44%) and conventional anaerobic fermentation (28%) [67]. Thereby, the mechanisms of EF will be further discussed in the section below.

### 3.1. Electro-fermentation (EF) – Enhanced L-lactic acid and bioelectricity production

EF is an electrochemically induced technique that regulates electron flux in both working electrodes (anode/cathode) and microbial augmentation, directing redox reactions toward specific products at a higher rate [67,68]. In this context, the bioelectricity generated by polarized working electrodes is not the main energy source but is instead a product of interest and triggers the fermentation process driving toward an imbalanced intracellular oxidation-reduction potential (ORP) condition [69]. The intracellular ORP is particularly important as it controls enzyme synthesis and gene expression in the electroactive bacteria, thus impacting the whole metabolic process and pathway. It can be estimated from the reduced/oxidized nicotinamide adenine dinucleotide (NADH/NAD^+^) ratio due to intracellular redox homeostasis [70]. Variations in the NADH/NAD^+^ ratio of the system influence the direction of electron flux, which will be bidirectional and thereby help resolve the shortcomings of conventional fermentation for desirable product yields. Electron dispersion at the anode tends to reduce the NADH/NAD^+^ ratio, resulting in compensatory cellular regulation favoring pathways for NADH replenishment. Subsequently, this mechanism further elongated the oxidative and reductive conditions in the EF system and promoted the formation of ATP molecules for cell division and anabolism, which results in a higher yield of products. Choi and Sang [71] revealed increased biochemical production yield in response to the additional NADH through cellular regulation.

**Figure 7.**
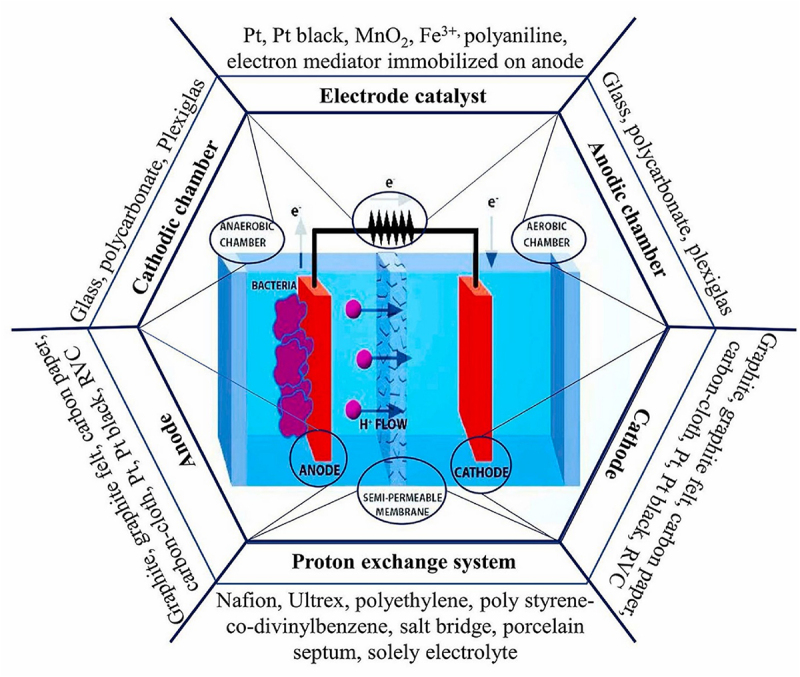
Basic components of microbial fuel cells and the materials used for structure [36].

Literature related to the virtues for the co-production of biochemicals and bioelectricity using EF system showed an increasing trend with expanding research outputs, leading EF technology a potential methodology for biorefinery [72,73]. An overview of the basic components comprising an EF system is mandatory to understand the significant role played by biofilm-producing microbes in the EF system. The typical EF system is assembled in a dual-chamber design consisting of an anodic and cathodic chamber divided by a PEM (Figure 3). Other designs, including single-chamber and stacked EFs, are also retrievable from the literature [74,75]. Figure 7 depicts the tenable materials that can be employed in the fabrication of the EF system. The anodic chamber is made up of one of the two primary components of EFs, which is referred to as an anaerobic chamber, signifying the critical role of exoelectrogens and electron mediators in it. When subjected to an appropriate anaerobic condition, exoelectrogens in the anode function as biocatalysts, oxidatively decompose organic substrate or hydrolyzate into simple molecules or biochemicals. This results in the generation of electron-proton pairs, which are later transferred to the cathodic chamber through the external circuit and PEM, respectively. As the electrons travel along the external circuit, inducing the current flow in the connecting wire, thereby producing bioelectricity [76]. Generally, aerating the anodic chamber is prohibitive, as it could retard the anaerobic performance of exoelectrogens in the EF system. This is because the presence of oxygen can result in the stripping of dissolved CO_2_ from the anode chamber, elevating the pH and inducing alkaline conditions. The pH shift may detrimentally affect the microbial community and electrochemical reactions, hurdling EF performance. Moreover, excessive aeration can lead to mechanical agitation and disruption of biofilm formation on the anode surface, reducing microbial activity and hindering electron transfer processes [77].

Contrarily, the cathodic chamber is referred to as an abiotic chamber, comprising electron acceptors for the electron reduction process [36]. Potassium ferricyanide (K_3_[Fe(CN)_6_]) is widely accepted as the electron acceptor, which attracts and dissipates the electron generated in an anode by reducing its Fe (III) ion to Fe (II) ion before reacting with the proton migrated from PEM. In terms of power generation, K_3_[Fe(CN)_6_] is an excellent electron shuttle, facilitating the transfer of electrons from the anode to the cathode, which enhances the electrochemical activity and performance of the EF system. This electron transfer stimulates the metabolic activity of exoelectrogens by balancing the redox reactions in the system [78]. Farah et al. [79] revealed that the power output attained are 8.33 W/m^3^ and 0.06 W/m^3^ for K_3_[Fe(CN)_6_] and phosphate buffer as catholyte, respectively, highlighting potassium ferricyanide possess superb catalytic activity. However, the utilization of K_3_[Fe(CN)_6_] is limited to large-scale applications due to poor reoxidation, which demands the catholyte to be frequently replaced. Further, the long-standing function of the system can be affected by the diffusion of ferricyanide ions across the PEM into the anodic chamber, resulting in aeration anodic chamber, which retard the metabolic activity of exoelectrogens [80]. Then, oxygen is employed as an electron acceptor owing to its availability. Such a process only yields water as the final product, signifying its positive environmental benefit, but oxygen possesses limitations of limited diffusivity (~2 × 10^−5^ cm^2^/s) and low solubility (2–4 mM) in water [81]. Considering the trend of miniaturization and integration for future EF systems, potassium permanganate (KMnO_4_) is one possible candidate oxidant due to its characteristics, including high oxidation ability, large redox potential, and high regenerability. KMnO_4_ is a potent oxidizing agent with a high standard reduction potential that can readily accept electrons from the anode, which enhances the electron transfer efficiency in the EF systems and promotes higher power generation [82]. KMnO4 has been applied successfully to yield an output voltage of 3003 mV, which conveniently powers low-voltage appliances [83]. In addition to the type of electron acceptor, catalyst activity is also a crucial factor in the performance of the EF cathode [84]. Giordano et al. [85] further boosted the power output of the EF system from 70 mW/cm^2^ to 115 mW/cm^2^ by applying 0.1 M sulfuric acid (H_2_SO_4_) as a supporting catholyte. This result underlined the positive effect of adding H_2_SO_4_ as a supporting catholyte in the EF cathodic chamber on the catalyst activity and electrochemical output [85]. The addition of H_2_SO_4_ as a supporting electrolyte improves the overall ionic conductivity of the system for electron transfer between cathode and anode as H_2_SO_4_ dissociates in water to release hydrogen ions (protons) and sulfate ions. The presence of hydrogen ions in the catholyte facilitates proton conduction from the cathode to the anode through the electrolyte, enhancing the electrical conductivity and open-circuit voltage. In fact, proton transfer is essential to the overall electrochemical reaction occurring in the EF system [82]. Besides, the sulfate ions generated from the dissociation of H_2_SO_4_ can act as the ion exchange sites of PEM due to sulfate ions approaching the behavior of sulfonic groups in Nafion®, increasing the mobility of protons through the membrane [85].

Regarding electrode material selection, chemical stability, biocompatibility, and conductivity are desired properties for optimizing EF performance [86]. Although benchmarked with its conductivity and arc erosion resistance, copper is not an ideal electrode material due to its antibacterial features. During anodic reactions, copper ions released from the anode will participate in redox reactions with the protons and generate reactive oxygen species (ROS), such as superoxide ions and hydroxyl radicals. The generated ROS can cause oxidative stress within exoelectrogen cells, damaging cellular components and disrupting the microbe cell membrane, leading to leakage of cell contents and ultimately cell death. Consequently, high copper concentrations can result in oxidative damage and impede microbial growth [87]. Thereby, non-corrosive and less harmful stainless steel and molybdenum steel mesh provide an excellent alternative to copper for electrode purposes in the EF system. Similarly, carbon-based materials, including graphite, carbon cloth, carbon felt, and carbon nanotubes, are among the most widely used electrode materials with high adaptability and excellent electrical conductivity [88]. Other than conductivity, the choice of electrode materials significantly influences the biofilm formation process and direct electron transfer performance of exoelectrogens to the anode. In EF, the exoelectrogens or bacterial attachment to and biofilm formation on the anode surface is crucial for the efficient biological transfer of electrons between the exoelectrogens and anode [89]. Biofilm is an extracellular polymeric substance (EPS) encased, surface-adhering microbial community. EPS is a self-produced matrix of exoelectrogens comprised polysaccharides, proteins, nucleic acids, and other biomolecules that surround the biofilm to provide structural support and protection to the biofilm community, which is an advantage for exoelectrogens’ survival. In theory, the formation of biofilm on the anode involves several steps: (1) exoelectrogens present in the anodic chamber initially come into contact with the anode surface as anode possess conductive properties and availability of electron donors, which is suitable for microbial attachment; (2) exoelectrogens begin to adhere to the anode surface via weak and reversible interactions which facilitated by factors such as van der Waals interactions, hydrophobic interactions, and electrostatic forces; (3) Upon exoelectrogens adhere to the anode, they will secrete EPS that holds the microbial cells together and provides structural stability to the biofilm; (4) When biofilm developed, exoelectrogen cells within the biofilm proliferate and undergo cell division, resulting in an increment in the population within the biofilm; (5) Within the biofilm, synergistic relationship develop between exoelectrogens, allowing for the electron transfer, exchange of metabolites, and enhanced biofilm functionality; (6) Over time, channels and void spaces may form within the biofilm as additional EPS and exoelectrogen cells are produced, stimulating the movement of metabolic by-products, nutrients, and electron transfer [90]. In this respect, the preferred characteristics of anode materials for efficient biofilm formation and development include good surface charge, rougher surface, hydrophilicity, and affinity for EPS components [89]. Among electrode materials, carbon felt and carbon nanotubes are the preferred material owing to the presence of microscopic irregularities, such as cervices and pores, aggrandizing structural integrity and biofilm stability. Furthermore, complementary charge characteristics of carbon nanotubes offer an excellent electrostatic interaction between the charged surface of the anode and exoelectrogens, facilitating microbial adhesion and subsequent biofilm development [88]. Nonetheless, the influences of electrode materials and arrangement on the power output will be discussed in Section 4.2.

**Figure 8.**
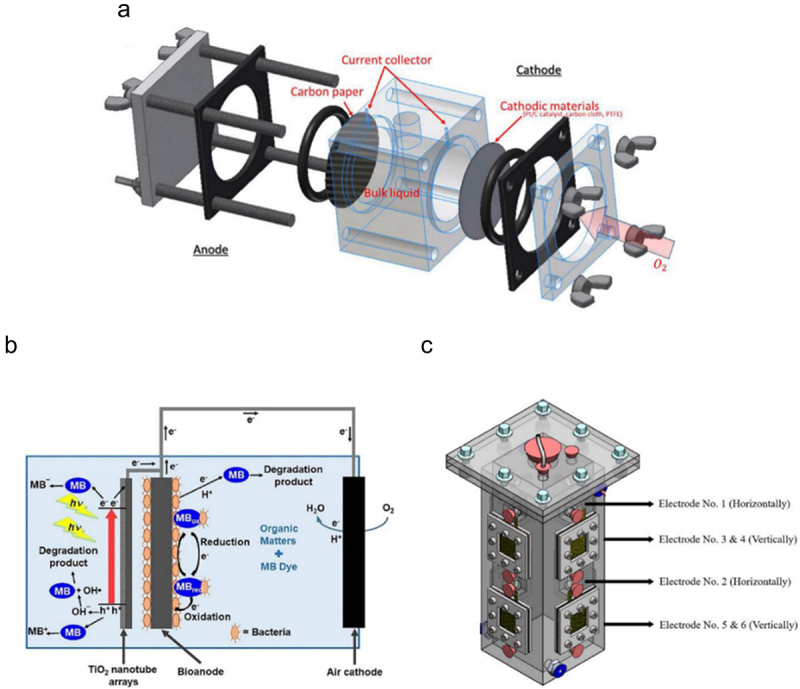
(a) Single-chamber air-cathode microbial fuel cell reactor [93], (b) single-chamber membraneless microbial fuel cells operating on the open-air cathode [92], and (c) single-chamber microbial fuel cells operating on stainless steel mesh painted by acrylic-based graphite [94].

Employing a dual- or single-chamber EF system relies heavily on the proportion and compatibility of the final products formed at the counter electrode. Precisely, owing to good scalability and irreplaceable simplistic design, single-chamber systems depict greater up-scaling convenience [91]. In most wastewater remediation cases, only an anodic chamber is mandatory, with the cathode exposed directly to the air. Hence, the inclusion of PEM can be excluded from this design, lowering the manufacturing cost [92]. As shown in Figure 8 (A), the anode and cathode are positioned at each end of the tube, with only the former covered by a flat plate, indicating the simplest design for a single-chamber system. Operationally, the anodic chamber houses the organic substrate and exoelectrogens, while oxygen from the air is constantly supplied to the cathode through diffusion, which yielded a maximum power density of 1320 ± 50 mW/m^2^ by using sodium acetate as the substrate [93]. Lee et al. [92] came up with an air-cathode MFC design having the carbon felt anode inside a cylindrical chamber, while the wet-proof platinum-coated carbon cloth air-cathode is positioned on the exterior of the device for wastewater remediation (Figure 8(B)). The authors revealed that 82.79% of methylene blue dye in the wastewater was successfully degraded while simultaneously generating bioelectricity with a current density of 4571.43 mA/m^2^ over 3.5 h of operation under simulated solar light illumination [92]. In another study, a cubic structure single-chamber air-cathode MFC was developed by applying six acrylic-based graphite-coated stainless-steel mesh as anode and six activated carbon-coated stainless-steel mesh as cathode, which is illustrated in Figure 8(C) [94]. This study indicated the importance of surface modification on stainless steel mesh to increase the overall power output of the system by 50.22%, yielding a maximum power and current density of 463.88 mW/m^3^ and 1991 mA/m^3^, respectively [94]. Nevertheless, besides the aforementioned merits of the air-cathode single-chamber system, the application of this system is still hindered by some setbacks. Complexity for air-cathode manufacturing is considered a significant setback as diffusion layer or noble metal catalysts, including platinum and argentum, are required to enhance the reduction performance. The increased complexity and associated costs can pose challenges for large-scale applications. Additionally, air-cathode can be degraded due to several factors, such as biofouling, chemical reactions, or mechanical stress. Regularly exposed to oxygen-rich environments, chemical reactions will take place at the cathode surface to produce ROS, leading to the degradation of the cathode surface and reducing the cathode’s lifespan [82].

**Figure 9.**
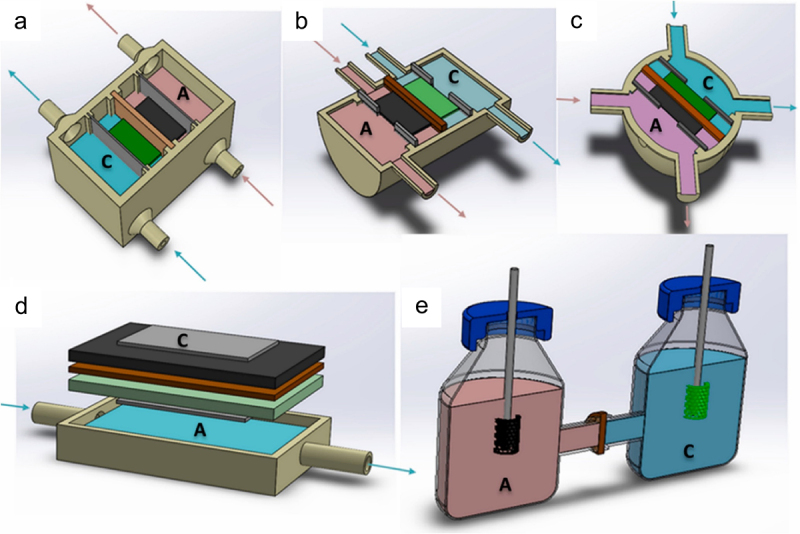
Diverse configurations for membrane-based microbial fuel cells. (a) Cuboid double-chamber MFC, (b) cylindrical double-chamber MFC. (c) spherical double-chamber MFC, (d) flat-plate double-chamber MFC, and (E) H-shape double-chamber MFC. In all designs, the “A” and “C” indicate anode and cathode, respectively [98].

Recently, multi-chamber, also known as stacked EF systems, have been explored as a potential design configuration for enhanced efficiency in biochemical production and electrical power capacity. Notably, the power capacity can be increased by connecting multiple individual unit chambers in either parallel or series configurations. Halim et al. [86] reported an 87% chemical oxygen demand removal efficiency with an optimal power density of 115.94 mW/m^2^ was attained in a parallel-wired stacked EF system. In a different study, a serially stacked EF system comprising graphite felt as the primary material for working electrodes was proposed by Kim et al. [95], which yielded 3.3 V as the maximum voltage, realizing higher efficiency up to 16.5 times (boosted voltage from 0.2 V to 3.3 V) compared to single EF system. Coincidentally, a declination in power output was discerned over time in both configurations. This was mainly attributed to ionic short-circuiting and voltage reversal. With only a single anolyte and catholyte input, organic overloading could occur due to inefficient flow distribution. Accumulation of organic substrate or deposition of non-conductive materials on the electrode surfaces can increase internal resistance of the EF system, leading to electrode fouling and voltage reversal. This further results in biofilm decay and reduced the electron transfer efficiency [96]. In view of this, the conventional configuration, the dual-chamber EF system is preferred for co-production of biochemicals and bioelectricity. Dual-chambered systems with membranes are applied when the optical purity of the bioproducts, such as L-LA and succinic acid is emphasized [97]. Similar to the previous genre, dual-chamber EF systems also come in diverse designs after years of development. From Figure 9 (D), the anodic and cathodic chambers of the dual-compartment EF system are assembled in a flat-plate design, in which the electrodes are stacked into a single layer divided by a PEM. Such design impelled an enhanced proton transfer with the closely packed electrodes, yielding a maximum open circuit voltage and power density of 637 mV and 232 mW/m^3^, respectively [99]. In addition, Gebreslassie et al. [13] proposed a H-shape dual-chamber EF system with Nafion® 117 PEM salt bridge completing the circuit and sustaining the electrical neutrality of the device (Figure 9(E)). The authors revealed that the dual-chamber EF system can be a promising integrated reactor process for the co-production of hydrogen and bioelectricity by achieving a hydrogen yield of 110 mL/g and a power density of 182 mW/m^2^ [13].

Aside from the physical design parameters, the biocatalyst inoculated to the anode surface also imparts a significant impact on the bioelectricity generation potential and biochemical conversion efficiency of the EF systems. The endowment of pure or mixed microbial communities for bioelectricity production depends on the system’s architectural and physiochemical environment, including electrode size, electrode materials, internal resistance, and acidity of the system [100]. Thus, the bioelectricity generation potential of a microbial culture cannot be compared with each other unless all physical and chemical parameters are similar. In the EF system, the capacity of microbes to decompose the organic substrates is primarily governed by the composition of the microbial community and its adaption [101]. The use of exoelectrogens as biocatalysts is favorable for generating electricity by organic substrate degradation [68]. Among exoelectrogen genera and species, the strain isolated from *Clostridiaceae*, *Shewanellaceaae*, *Geobacteraceae*, and *Pseudomonadaceae* genus, is of great interest phylum because of their capacity for extracellular electron transfer (EET) by direct electron transfer to the working electrode [102–104]. These exoelectrogens are usually negatively charged and generally known as Gram-negative microbes due to the presence of uronic acids and ketal-linked pyruvates in the cell plasma membrane, thereby supplying the anode with positive potential and divalent cations, including calcium and magnesium, which maximizes the scope of biofilm formation on the anode surface by involving electrostatic interactions [105]. Then, a negative potential is generated at the cathode to expedite the reductive reaction with the oxidizing agent by increased electronegativity in the system [68]. In terms of direct electron transfer, *Clostridium* sp. can catalyze EET to the working electrode by excreting ferredoxin as endogenous mediators, while gram-negative proteobacterium belonging to the *Geobacteraceae* genus uses pili as conductive filament for EET [103]. In addition, *Shewanella* sp. possesses both EET mechanisms, which use nanowires as conductive filaments and excrete riboflavin as endogenous mediators to catalyze bioelectricity production [106]. Mechanistically, the metabolism of the organic substrate by exoelectrogen drives the production of NADH within the cell. For the cell to retain reducing power, NADH will re-oxidize to NAD^+^ by abstracting electrons and biochemicals, such as L-LA, by using dehydrogenase enzyme and the cytochrome system comprising menaquinone/quinone pool, periplasmic proteins, MacA, outer membrane OmcE protein, and outer membrane OmcS protein. These cytochrome proteins facilitate the electron transports by a series of redox reactions spanning the inner cytoplasmic membrane across the periplasmic space until the electrons are conducted across the outer membrane to the anode through outer membrane cytochromes OmcE and OmcS. Further, the direct electron transfer also occurs within multilayers of cells through a dense network of appendages with metal-like conductivity known as bacterial pili/nanowires. The transfer is manipulated cell by cell until electrons are donated to the electrodes [107].

The details of pure microbial cultures used in the EF systems are provided in Table 1. Through pure microbial cultures, an insightful alteration in the process taking place during electron transfer and the complex behavior of individual species in mixed microbial cultures can be explored and studied. To date, exoelectrogenic *Geobacter sulfurreducens* have been gaining attention in the scientific community for their capacity to generate high power density [104]. Applying *Geobacter* strains in EF systems manifests a significant advantage, whereby the generated electrons are directly transferred to the electrode through pili while vying for the limited surface area on the anode, which yielded a power density of 325 mW/m^2^. Theoretically, electrons were transferred to the anode via the membrane-bound redox proteins, with the direct EET controlling the overall shuttling rate [120]. Furthermore, pure culture with exoelectrogenic *G. sulfurreducens* was also applied successfully to metabolize carboxymethyl cellulose for the generation of acetic acid with a concentration of 77 mg/L and bioelectricity with a maximum power density of 1146 ± 28 mW/m^2^ in a dual-chamber EF system by Jiang et al. [115]. In contrast, *Shewanella* strains are relatively weaker in the aspect of generating electricity from the organic substrate. Evidently, only partial oxidation of sodium lactate into acetate can be provoked by *Shewanella oneidensis*, resulting in a lower overall amount of generated bioelectricity with a power density of 89.40 mW/m^2^ after 6 operation days. This can be attributed to the anaerobic nature of organic substrate metabolism and the non-central extracellular feature of the intermediates [111]. However, Dai et al. [112] revealed that a higher power density of 578 mW/m^2^ was attained using exoelectrogenic *S. oneidensis* in a dual-chamber EF system with bamboo fermentation effluent as the substrate for 8 operation days. The increment of the power capacity is significantly affected by the operation period as *S. oneidensis* will secrete flavins, including flavin mononucleotide (FMN), flavin adenine dinucleotide (FAD), and riboflavin in the concentration of 100–500 nM after 1 week of operation [98]. The excreted flavins will then act as redox mediators to promote electron transfer from microbial cells to the electrode surface. The FAD and FMN are mainly involved in intercellular metabolism by mediating the oxidation of NADH and reduction of NAD^+^ to maintain the imbalanced intracellular ORP. At the same time, riboflavin is proposed to interact directly with outer membrane c-type cytochromes as a bridge between the working electrode and the cell for EET [121]. The redox mediators can be reduced or oxidized by the exoelectrogens and then be recycled electrochemically at the working electrode [122]. Indeed, García-Mayagoitia et al. [108] concluded that the flavins excreted by the *Bacillus subtilis* strain and work as redox mediators were shown to enhance the bioelectricity production in the MFC system up to 105 mW/m^2^.

**Table 1.** A comparison study of pure culture and co-culture for electrofermentation system on L-lactic acid and bioelectricity production.

Electroactive bacteria strain	Biomass/Anolyte	Working potential^a^ (V)	Redox mediator	Intended product		References
				L-lactic acid (g/L)	Bioelectricity	
**Pure culture approach**						
*Shewanella oneidensis*	Glucose	−0.30	Riboflavin	-	138,181^b^	[102]
*Lactiplantibacillus plantarum*	Kale hydrolysate	0.20	-	1.13	752.14^b^	[97]
*Lactococcus lactis*	Glucose	0.35	-	-	212.00^c^	[73]
*Bacillus subtilis*	Pharmaceutical residual water	0.20	-	-	105.00^c^	[108]
*Lactobacillus pentosus*	Diary whey	0.55	Yeast extract	-	5.04^c^	[109]
*Clostridium cochlearium*	Glucose	0.19	Thiamine Riboflavin	-	5,300.00^b^	[103]
*Bacillus megaterium*	Activated sludge from wastewater with acetate	0.40	Exogenetic flavins	-	170.00^c^	[110]
*Shewanella oneidensis*	Sodium lactate	0.20	-	-	89.40^c^	[111]
*Shewanella oneidensis*	Bamboo fermentation effluent	0.30	-	-	578.00^c^	[112]
*Lactobacillus bulgaricus*	Diary whey	0.20	-	19.50	288.12^c^	[113]
*Bacillus tequilensis*	Glucose	0.17	-	-	407.96^c^	[114]
*Geobacter sulfurreducens*	Cellulose	0.30	-	-	1146.00^c^	[115]
**Co-culture approach**						
^d^*Bacillus subtilis*^e^*Saccharomyces cerevisiae*	Glucose	0.50	-	-	602.00^b^	[116]
^d^*Lactococcus latis*^e^*Lactobacillus acidophilus*	Glucose	0.20	-	-	415.00^c^	[117]
^d^*Bacillus tequilensis* ^e^*Pseudomonas aeruginosa*	Glucose	0.17	-	-	8,159.27^c^	[114]
^d^*Shewanella oneidensis*^e^Engineered *Saccharomyces cerevisiae*	Glucose	0.20	-	7.50	123.41^c^	[118]
^d^*Shewanella oneidensis*^e^*Klebsiella pneumonae*	Glycerol	0.15	-	-	10.00^b^	[119]

^a^Working potential: working electrode potential versus standard hydrogen electrode (SHE) potential.

^b^Electric output in terms of current density with SI unit mA/m^2^.

^c^Electric output in terms of power density with SI unit mW/m^2^.

^d^Main electroactive bacteria strain.

^e^Supplementary electroactive bacteria strain.

In addition to the self-secreted (endogenous) mediators by the exoelectrogens, Shirkosh et al. [102] explored bioelectricity production through EF from a glucose medium by using *S. oneidensis* with exogenetic riboflavin. Such endowment prompted an enhanced bioelectricity capacity and lifespan of the system, which yielded a maximum current density of 138,181 mA/m^2^ with a prolonged operating duration of 50 days. These results are nearly 445% better than the values obtained without exogenetic riboflavin, indicating that the exogenetic riboflavin is essential for the survival of *S. oneidensis* in anaerobic conditions by offering the ability to access insoluble electron acceptors [102]. This is attributed to the relatively slow production rate of endogenous mediators by *S. oneidensis* and will only produce limited concentrations in the stationary phase when the microbial cells are lysing [98]. Thus, the endowment of exogenic riboflavin in the culture medium can boost the electron transfer process by undergoing reversible redox reactions, shuttling between its reduced (riboflavin semiquinone) and oxidized (riboflavin) forms. The riboflavin present in the extracellular environment will interact and bind with the outer membrane c-type cytochromes of exoelectrogens, forming a complex called riboflavin semiquinone with an electron attached to it. Then the riboflavin semiquinone diffuses from the exoelectrogen cells to the electrode surface. Once at the electrode surface, the riboflavin semiquinone transfers the electron to the electrode, completing the electron transfer pathway and shifting back to its oxidized form. This transfer is considered a direct electron transfer through the interactions of the mediator with the electrode, facilitating electron exchange [123]. Another study reported bioelectricity production from activated sludge via EF using *Bacillus megaterium* with exogenic flavins. The optimum power density of bioelectricity was enhanced by 4.6-fold and reached approximately 170 mW/m^2^ compared to that without flavins addition under similar operating conditions [110]. Similar results were also found in the study of Schwab et al. [103] that the bioelectricity with a maximum current density of 5300 mA/m^2^ could be generated from glucose by *Clostridium cochlearium* isolated from the mouse gut after 5.2 h of operation duration with the addition of exogenic thiamine and riboflavin.

**Figure 10.**
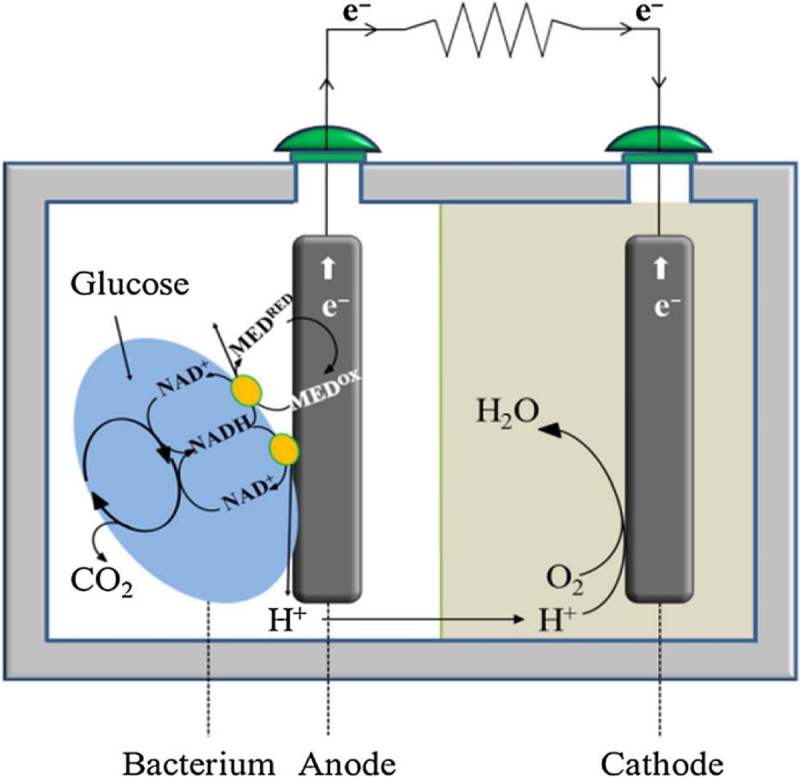
Schematic principle of a microbial fuel cell with lactic acid bacteria as biocatalyst for co-production of L-lactic acid and bioelectricity [126].

To date, most species studied for bioelectricity generation in EF systems are Gram-negative microbes. Interestingly, few Gram-positive LAB strains from *Lactobacillus*, *Lactococcus*, *Lactiplantibacillus*, and *Bacillus* genus are recognized to be both electroactive and able to metabolize a broad range of carbohydrates to valuable L-LA [73,97,108,109]. The LAB strains isolated from the abovementioned genus are considered excellent producers of high-purity L-LA as they contain only the *ldhL* gene that secretes L-LDH enzymes, which induces the formation of pure L-LA [124]. In this regard, Tejedor-Sanz et al. [97] demonstrated that both rare sugar (xylose and glucose) in the kale hydrolyzates can be fermented simultaneously to obtain a L-LA yield of 1.13 g/L and bioelectricity with a maximum current density of 752.14 mA/m^2^ by using *Lactiplantibacillus plantarum* at optical density (OD_600_) of 1.0 in a H-shape dual-chamber EF system. Under the same OD_600_ of *Lactobacillus bulgaricus* cells, the EF process for the diary whey was optimized to achieve the intended product in terms of L-LA yield of 19.5 g/L and bioelectricity with an optimum power density of 288.12 mW/m^2^ [113]. Similarly, an EF system with *Lactobacillus lactis* DLP27 strain as anodic-respiring bacteria has been explored with a bioconversion yield of up to 212 mW/m^2^ from glucose medium for bioelectricity production [73]. This is mainly due to the fact that *L. lactis* can excrete liposoluble quinones (2-amino-3-carboxyl-1,4-naphthoquinone) to facilitate the EET by connecting the intracellular metabolism of the cell to extracellular redox reactions [125]. Mechanistically, LAB present in the electron-rich substrate, such as hydrolyzate comprising glucose molecules, will manipulate PTS to transport the glucose molecules to its cytoplasm for metabolism reaction. Once the glucose molecules are at the cytoplasm, LAB will proceed with either homolactic or heterolactic fermentation to produce L-LA as the end product. Glycolysis of glucose molecules drives the production of electrons within the cell, resulting in an imbalance of ORP level. For 100% coulombic efficiency, 24 electrons will be produced per glucose molecule. In order for the cell to maintain a balance ORP level, it will excrete liposoluble quinones as an endogenous mediator to shuttle the electron away from the cell to the electrode surface (Figure 10). In brief, LAB will metabolize the glucose molecules, abstracting L-LA and electrons. The electrons are then transferred from the LAB cell to the electron mediator and subsequently to the electrode, generating an electrical current in the external circuit of the EF system [97].

Although research is emerging in the pure microbial culture EF system, all these methods are extendable to fermenters and electroactive bacteria co-culture for supplementary benefits [118,119]. A comparative study has been reported on pure culture and co-culture for bioelectricity production from glucose medium as an anolyte in the EF process (Table 1). The yield of bioelectricity in terms of current density was enhanced by 141.92% with co-culture using *B. subtilis* as an electroactive bacteria and *Saccharomyces cerevisiae* as a fermenter [116]. This is mainly contributed by the syntrophic interaction among the bacteria strains, which offers an interspecies electron transfer (IET) between the bacteria, either directly via conductive pili or indirectly via diffusion of redox mediator [127]. In this case, *B. subtilis* excreted flavins as mediators via respiration to induce the EET in the anode and reduce the internal resistance of the EF system. Subsequently, *S. cerevisiae* transmits electrons through the flavins produced to reduce the resistance of electron transfer as well as a direct contact mechanism. Finally, the bioproducts produced by the decomposition of glucose by *S. cerevisiae* via metabolism served as an electron donor and carbon source for *B. subtilis*. The bioproduct consumption by *B. subtilis* in the biofilm through IET mechanisms facilitates the EF process and increases the purity of the intended products [116]. Further, Abdel-Gelel et al. [117] developed a co-culture EF system by utilizing a combined LAB strain of *L. lactis* and *Lactobacillus acidophilus* for bioelectricity production from a glucose medium. A pure culture EF system was conducted as a standard reference, where pure *L. lactis* and co-culture produced a power density of 152 mW/m^2^ and 405 mW/m^2^, respectively [117]. The presence of cyclic diguanylate monophosphate in the intracellular of *L*. *acidophilus* stimulates biofilm formation and promotes *L. lactis* to produce more liposoluble quinones for EET to minimize the internal resistance and increase the output voltage of the EF system [117].

### 3.2. CO_2_ capture and conversion

Associated with the EF reaction in the MFC system is the generation of hydrogen (H^+^) and hydroxyl (OH^−^) ions in the anolyte and catholyte, respectively, which creates a pH imbalance in the system as the H^+^ ions mass transfer is sluggish that will be accumulated in the anode causes anolyte acidification [93]. Experiments have shown that acidic or basic pH significantly decreases the current density, voltage efficiency, and resultant power output by detrimentally affecting the electroactive bacteria metabolism via *Le Chatlier*’s principle [128,129]. From an electrochemical perspective, pH imbalance also increases concentration overpotential for substrate oxidation and potential loss for the whole EF system [129]. Chen et al. [130] revealed that inorganic carbons (carbonic acid, H_2_CO_3_; carbonate ions, CO_3_^2-^; bicarbonate ions, HCO_3_^−^) produced through the reduction of nearly 100% CO_2_ generated during microbial respiration are indispensable in EF to offer ionic conductivity and retain stable pH conditions of the electrolyte. The CO_2_ generated in the system reacted with OH^−^ ions to form inorganic carbons, which became the highest concentration of anions in the catholyte. With an anion exchange membrane, the inorganic carbons were transported from the cathode to the anode to retain electroneutrality and catalyzed the diffusion flux of anion ions [93].

Aside from being applied successfully for CO_2_ capture, EF is adapted more toward CO_2_ fixation using various mechanisms. In the context of CO_2_ fixation using EF, the potential differences of the working electrodes act as a steering force to convert/reduce CO_2_ to value-added bioproducts due to CO_2_ being thermodynamically stable [131]. One such mechanism is electrochemical CO_2_ reduction to bioethanol through biological interventions that capture and convert CO_2_ [97]. Besides L-LA, short-chain carboxylic acid such as acetic acid formation also occurs due to the degradation of L-LA by the heterofermentative *L. plantarum*. The accumulation of acetic acid and L-LA creates a surge in maintaining the balance between anolyte and catholyte pH that manifest in the CO_2_ reduction and H_2_ formation via the reduction of H^+^ ions, thereby promoting bioethanol production in the presence of H_2_ as an electron donor by reducing the acetic acid with cathode poised potential between −0.7 and −0.9 V [97]. Biogas such as biomethane (CH_4_) synthesis was also perceived upon CO_2_ reduction in several research using EF [132,133]. CO_2_ reduction within the EF system toward biogas upgradation enhances product yield and lowers the process energetics. In general, volatile fatty acids and H_2_ are electron donors in converting CO_2_ to CH_4_. With the ability of H_2_ to implement as a carbon-free biofuel for the transportation sector, direct IET mediated by nanowires or pili of electroactive bacteria was proposed as an alternative to interspecies H_2_ transfer [134]. CO_2_ is reduced by direct electron transfer at the catholic chamber at a potential lower than −0.24 V vs SHE, with 94.14% of the applied potential converted into CH_4_ [133]. Methanogens assist in exploiting *in situ* produced H^+^ ions and electrons as a redox mediator for direct IET coupled to CO_2_ reduction toward increased CH_4_ synthesis involving less activation energy as a part of the electrocatalytic activity, which is an added benefit [135].

Alongside the production of bioelectricity, the production of medium carboxylic acids including butyric acid and caproic acid was also observed during the EF system operation using *Clostridium kluyveri* as catholic respiration bacteria at −0.6 V for CO_2_ fixation [136]. The simultaneous presence of CO_2_ and acetic acid in the EF system presenting *Clostridium* spp. can lead to longer chain carboxylates via the reverse β-oxidation chain elongation pathway (Wood-Ljungdahl pathway) by excreting the acetyl-CoA to promote elongation from acetic acid (C2) to butyric acid (C4), and then from butyric acid to caproic acid (C6) [137]. Notably, butyric acid and caproic acid possess high commercial values and are broadly employed as antimicrobial agents, feed additives, and flavor enhancers in the pharmaceutical and food industries [138]. Each final product has designated poised potential regulated by the applied potential for driving the bioelectrochemical synthesis of bioethanol/biogas/carboxylic acids via CO_2_ reduction. EF-MFC integration has depicted a significantly increased CO_2_ capture and conversion toward carbon neutrality in the total bioproducts production by slashing the GWP of the process [139,140].

## 4. Microfluidic microbial fuel cells

**Figure 11.**
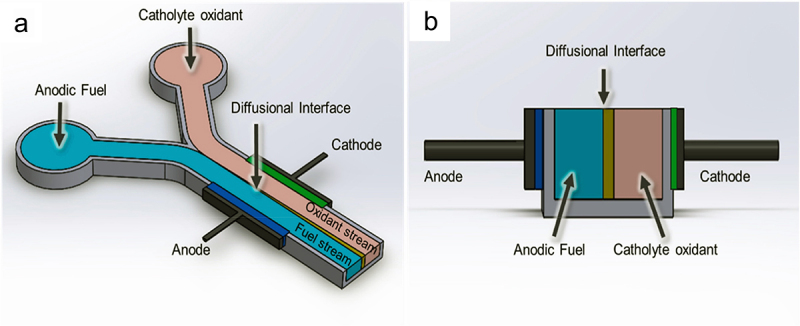
Schematic design of microchannel for membrane-less microfluidic microbial fuel cell: (a) top view and (b) cross-sectional view [98].

Advances in microtechnology and growing societal demand for portable devices that can operate for extended periods without recharging have paved a new route for the development of miniaturized electronic devices and materials for diverse industries, including environmental, biological, defense, and clinical applications [141,142]. From that perspective, a microfluidic microbial fuel cell (µMFC) is a promising alternative L-LA production platform and carbon-neutral energy source for bioplastic applications and portable microelectronic devices [143]. The idea of µMFC emerged from the conventional MFC with a total cell volume of 1–250 µL. Operating such devices in a microenvironment offers a quick response time to reactants, high surface area to volume ratio, robustness, and can be well staked or scaled up [144]. Nevertheless, most quintessential µMFCs are downsized MFCs, with poor energy efficiency being one of the major setbacks (Table 2). High internal resistance is acknowledged as the primary factor of inadequate power output, which is ascribed to the involvement of the membrane [149]. To realize the real-time feasibility of µMFC, co-laminar fluid flow arrays are being exploited, where immiscible anolyte and catholyte flow in the microchannel to make µMFC membrane-less which eliminates the external influence of inertial forces on the separation membrane while still consolidating the advantages of microfluidic flow for power generation [147]. The common channel configuration of membrane-less µMFC is depicted in Figure 11, whereas both electrolytes are separated by the diffusional layer created by laminar flow.

**Table 2.** An overview of some common challenges encountered in conventional microbial fuel cell technology.

Main obstacles	Description	Reference
High internal resistance	The involvement of PEMs or separators between the anodic and cathodic chambers introduced additional resistance to proton transport.	[145,146]
Limited scalability	Scaling up dual-chamber MFCs is challenging due to the increased complexity and potential issues associated with maintaining the PEMs.	[111]
Low coulombic efficiency	The separation of anodic and cathodic chambers by PEM can lead to side reactions or electron losses, reducing the overall efficiency of electron transfer.	[144,147]
Low power density	MFCs currently have lower energy conversion efficiency due to electron losses by high internal resistance.	[141]
Maintenance and system complexity	MFCs require regular maintenance to prevent membrane fouling, electrode degradation and system failures.	[141]
Membrane fouling	PEMs in dual-chamber MFCs are susceptible to scaling or fouling attributed to the accumulation of biofilms, salts, or other contaminants, lowering the membrane permeability and hindering proton transport.	[144,147]
Slow startup and lag phase	MFCs often require a startup period for exoelectrogens or microbial colonization and biofilm formation, leading to a delay in power generation as low cell density is inefficient for electron transfer.	[148]

An I-shape microchannel membrane-less µMFC with a total volume of 50 µL produced a maximum power density of 35,294 mW/m^2^ by *S. oneidensis* MR-1, which is circa 200% increment compared to membraned µMFC. The two streams are mixed by transverse diffusion alone [102]. In fact, the fluidic Reynolds (Re) number of the two streams in the microfluidic chamber, which contrasts the inertial force to the viscous force, is comparatively small that ranges between 10 and 100 and is typically considered as laminar flow [144]. Due to the nature of the laminar flow, the mixing of both streams is mainly restricted to a narrow interface and defined as diffusion across the mutual liquid–liquid interface between the two streams transverse to the direction flow. The liquid–liquid interface between the two streams in the laminar flow-based µMFC offers certain advantages over static membrane µMFC, including (1) promotes convective transport over diffusive transport so anolyte crossover can be eliminated as the amount of diffusion transverse to the direction of flow can be regulated with high precision by alteration of the anolyte and catholyte flow rate; (2) eliminating the water management issue caused by the separation membrane as it has to be hydrated all times to facilitate the transport of subatomic atoms; (3) can operate at elevated temperatures which enhances the bioelectrochemical reaction due to promote the microbial kinetics [148].

Owing to their modest size, µMFCs are compatible with several simple microfabrication techniques. Initially, non-polymeric materials including glass and silicon were utilized as starting materials for microfluidic device fabrication. Glass materials have remarkable biocompatibility and possess superior endurance to harsh conditions (high acid concentration and temperature) and are thereby preferred. However, non-polymeric-based µMFCs are commercially limited due to their micromachining complexity, high fabrication cost, and non-optical transparency of silicon [150]. Hence, polymeric materials such as polymethyl methacrylate (PMMA) and polydimethylsiloxane (PDMS) have gained considerable popularity in replacing non-polymeric materials for µMFC device fabrication due to their design flexibility, transparency, biocompatibility, durability, and low cost [150].

Latterly, some well-developed fabrication techniques demonstrated for lab-on-chip and electronics applications have been effectively adapted to µMFC fabrication. Photolithography, soft lithography, xurography, and paper-based µMFC are a few types of fabrication techniques among them. In general, photolithography is applied to non-polymeric materials in which a thin film of photoresist (typically SU-8) is spin-coated on glass or silicon wafer and covered with an image mask, then exposed to ultraviolet (UV) light for soft baking. Chemical vapor deposition is used to remove the exposed layer of photoresist, resulting in mold [145]. But this technique has limitations like non-suitability for pattern creation on non-planar surfaces and the requirement of expensive equipment. Thus, for polymeric-based µMFC, soft lithography was established as an extension of the photolithography technique as it is flexible and economical [150]. In addition, soft lithography utilizes a patterned elastomeric polymer as mold, allowing polymers, organic monolayers, and gels to be used for microchannel fabrication [151]. Briefly, a layer of image mask and the photoresist is coated on the elastomeric polymer. The masked polymer is then subjected to UV light, and the unexposed part is removed by immersing it in the SU-8 developer solvent. The remaining part is employed as mold, and polymeric materials such as PMMA and PDMS are poured into the mold and rectified to obtain the intended microstructure [152]. Luo et al. [147] soft lithographically fabricated a 10 µL Y-shaped membrane-less dual-chamber µMFC using PMMA and SU-8 photoresist. Using 50 mM potassium ferrocyanide as the catholyte and 10 mM lactate as an anolyte, the authors observed a maximum power density of 360 mW/m^2^, with 65% electrochemical efficiency [147]. The PMMA micromachining combined with the soft lithography technique was used to fabricate a 350 µm depth microchannel for dual-chamber µMFC, which yielded a maximum power density of 343 W/m^2^ [153].

Paper-based µMFCs have also been developed to fabricate the microchannel by a paper matrix, which provides significant advantages. To this extent, the natural wicking action of paper-based microchannel offers rapid adsorption of exoelectrogen fluid that urges adhesion of exoelectrogen cells to the working electrode and thereby significantly reduces the retention time of the bioelectrochemical reaction [154]. In addition, paper possesses the capability to flow fluids through capillary action mechanisms, offering passive liquid transport, and eliminating the requirement of external ancillary devices for feeding the electrolyte to the µMFC environment [155]. Several researches have been conducted to use the paper-based µMFC approach to develop membrane-less µMFC. Nath et al. [148] discovered a disposable and inexpensive membrane-less µMFC fabricated on a Whatman filter paper with a T-shaped microchannel. With this system, the devices were shown to hold exoelectrogenic *Escherichia coli* for 180 min, producing a maximum current density of 450 mA/m^2^, and a maximum power density of 27 mW/m^2^. Rewatkar and Goel [154] proposed a 250 mm2 Y-shaped paper-based microchannel that produced a maximum power density of 145 mW/m^2^. Further, an optimal power density of 240 ± 50 mW/m^2^ was attained using Whatman, grade Fusion 5 paper fabricated µMFC. This study revealed that the paper-based µMFC can produce similar power output as the µMFC operated with an external syringe pump using similar working electrodes and electroactive bacteria strain [156].

### 4.1. Integrated with EF (Advancements in microfluidic MFC operations-LLA production and electricity) − 3D printing technology

Recently, additive manufacturing technology based on 3D printing has imparted significant advances in the fabrication and design of complex structures through the Fused Deposition Modeling (FDM) technique [146]. This technology offers new potential for designing the highly personalized structure and the fabrication of final products through the sequential deposition of thin layers using the extrusion of thermoplastic polymeric fiber [157]. The rising concurrence of adopting the 3D printing system over the conventional device fabrication techniques has resulted in numerous benefits including fabrication of complex geometry with exceptional resolution in a short period, customization, outstanding design flexibility, and material economy [158]. With these unique and immense versatility properties, FDM can expand the range of novel µMFC and electrode architectures. Freyman et al. [159] have harnessed the 3D printing technology for making bacterial graphene-polycarbonate (PC) electrodes for direct electron transfer using electroactive bacteria *S. oneidensis* ATCC70050 for lysogeny broth valorization. To confirm and facilitate heterogeneous electron transfer, tryptic soy broth as a growth medium was included in the printing material for 3D-printed graphene-PC electrode fabrication to enhance the electroactive bacterial growth. The experimental data reported that the 3D printed graphene-PC electrode possesses lower charge transfer resistance (70.65 Ω) compared to the same material solid electrode (177.1 Ω) and produces a volumetric power density of 8.5 W/m^3^ attributed to better diffusion and higher surface area of the 3D printed graphene-PC electrode [159].

Moreover, Bian et al. [160] revealed that 3D printing could offer the precise design of 3D microporous lattice structured electrodes with UV-curable resin as printing material. Further surface modification of 3D printed polymeric electrodes with copper plating could enhance the power density by 12.3-fold compared to copper mesh electrodes. Notably, the 3D microporous lattice structure offers excellent biocompatibility and a large surface area to *S. oneidensis* MR-1 for bioproduct conversion and biofilm development, while the metal coating facilitates direct electron transfer [160]. Similarly, a rectangular mesh 3D printed PLA electrode has been developed to produce a stable power output (43 ± 1 µW) for almost 840 h [161]. A further example of material extrusion applied to µMFC device fabrication was reported by Rewatkar et al. [141]. In this work, the authors employed FDM 3D printing to manufacture a Y-shaped microchannel. The 3D-printed microchannel represented the working chamber for anolyte valorization and catholyte reduction. The 3D printed microchannel comprised an enduring and flexible rectangular holder made of conductive acrylonitrile butadiene styrene (ABS) based filament. Interestingly, conductive ABS filament showed better sensitivity and transferability in electrons; with this advantage, it was selected as the material for 3D printing of the microchannel [141].

**Figure 12.**
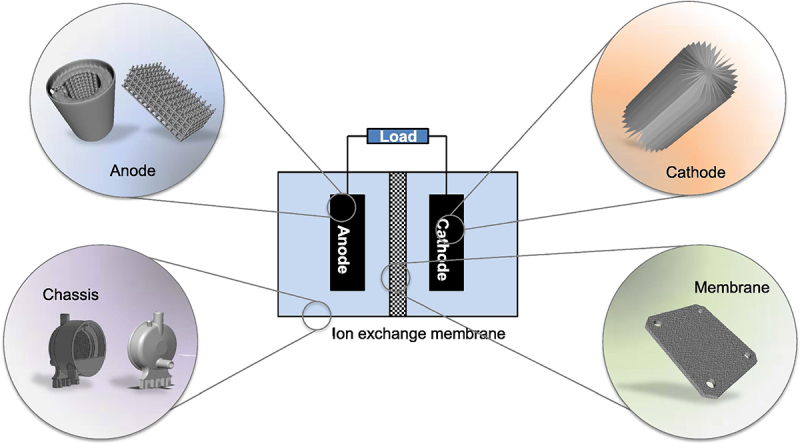
3D printable components of a microbial fuel cell [161].

Regarding the employment of FDM technology for the manufacture of ion exchange membrane (IEM), You et al. [161] constructed a 3D printed membrane using Gel-Lay filament, a filament composed of rubber elastomeric polymers and polyamide (Figure 12). The power output from µMFC with a 3D printed membrane was comparable to a commercial proton exchange membrane (240 ± 11 µW vs 177 ± 29 µW). Intriguingly, polyamide possesses strengths of low coefficient of friction which reduces the internal energy loss caused by the proton transfer to the cathode chamber via IEM. Additionally, the polymeric 3D-printed membrane also resists biofouling due to good fatigue and impact strength properties [161]. Theodosiou et al. [162] compared 3D printed membrane electrode assembly with a commercial IEM. The 3D printed membrane from air-dry clay materials exhibited twofold higher power outputs (130 µW vs 66 µW) for 11,760 h. This is attributed to the groove and emboss structure of the 3D printed membrane, resulting in a larger surface area than commercial IEM. Patterned 3D-printed membranes can exhibit reduced concentration polarization and improved ion exchange transport performance while alleviating fouling [163].

### 4.2. Electrode material and arrangement

The achievable current and power output of µMFC systems are reliant on the electrode material and architecture, which directly influences the biofilm formation, catholyte reduction, and electron transfer of the system. Inevitably, attaining a higher power performance µMFC requires a suitable electrode material with a large surface area, good biocompatibility, good electron conductivity, low electrical resistance, good surface hydrophilicity, and chemical stability properties for microbial adherence [164]. Recently, carbonaceous-based material has been extensively used for the construction of electrodes for µMFC systems [165]. Nonetheless, the commercial carbonaceous electrode showed a smooth surface with limited biocompatibility and electrochemical activity. Notably, a variety of strategies for carbonaceous electrode surface modification have been proposed. Chen et al. [166] developed a candle-soot-coated carbon cloth electrode by inoculating *Aeromonas hydrophila* in the MFC system. The candle soot coating altered the hydrophobic surface of the carbon cloth electrode to hydrophilic and offers a natural 3D mesoporous ordered structure for biofilm growth. The modified electrode exhibited the lowest internal resistance of 619 Ω with the optimal power density of 19.8 ± 0.2 mW/m^2^, which was 48.48% higher than the bare electrode under the same operation conditions [166]. The formation of biofilm on the electrode of µMFCs presents some significant advantages, whereby the generated electrons are directly transferred from the microbial cell to the electrode. This is attributed to the biofilm matrix providing a structured environment where the microbial cells can establish physical contact with the electrode surface, improving electron transfer efficiency and power generation. Additionally, biofilm improved the resistance of microbial cells to toxicity by functioning as a physical barrier to reduce the direct contact between toxic substances and microbial cells, exhibiting increased tolerance to toxic substances compared to planktonic suspension cells [98]. In another study, Liu et al. [167] chemically modified carbon paper electrodes with 0.5 M sulfuric acid for the µMFC system. The authors revealed that the modified carbon paper displayed a rougher surface with deep ditches as the sulfuric acid etched the carbon fibers to increase the surface area of the electrode for the biofilm formation. The modification reduced the anodic charge transfer resistance with a maximum volumetric power density of 235.6 mW/cm^3^, which was a 1.58-fold increment than the unmodified carbon paper [167].

Further, carbonaceous-based nanomaterials such as carbon nanotubes (CNT) and multi-walled carbon nanotubes (MWCNT) were modified to enhance anodic biofilm formation. The CNT composites can improve cellular adhesion electrostatically and increase electrode surface roughness [168]. Bandapati et al. [169] realized the MWCNT-coated HB pencil graphite (MWCNT/PG) electrode using the dip coating method. The performance of MWCNT/PG showed a higher current density (4,605 µA/cm^2^) than the bare PG electrode. Besides, the MWCNT/PG displayed a more extensive biofilm of microbial attachment and cyclic volumetry, which indicated that MWCNT offers good electric contact between the active site of the microbial and the PG surface [169]. A reduced graphene oxide nanosheet-coated carbon cloth anode has been applied successfully for bioelectricity production in the MFC system with a maximum power density of 10.8 ± 0.19 mW/m^2^. Reduced graphene oxide nanosheets with superior electrical properties and rich hydrophilic functional groups had established a strong electrochemical performance in the MFC system [170]. Moreover, the usage of granular electrodes for MFCs is considered a cost-effective assay for providing high surface areas, which is advantageous for achieving high power output [171]. Activated carbon granules as soluble electron acceptors attached to *G. sulfurreducens* are capable of extracting electrons from acetate and storing the electrons in the granules. Briefly, the granular activated carbon behaves like a capacitor where the protons produced from anolyte oxidation will store in the bacterial biofilm on the granules along with charge and granules are discharged when in contact with the current collector. Consequently, during the discharge of granular activated carbon, protons are also released along with electrons, leading to localized high conductivity and negligible ohmic losses due to low electron transfer resistance [171].

Notably, the use of metal electrodes is on the rise due to possessing good mechanical strength and higher electrical conductivity compared to carbonaceous-based electrodes. Evidently, an interesting outcome was perceived during the use of the molybdenum electrode. This study found that the molybdenum electrode produced a comparable power density of 1296 mW/m^2^ with an internal resistance of 62.3 ± 3 Ω, which is a 50-fold increment compared to the carbon paper electrode. This is mainly attributed to the appearance of the cleft-like alligatoring surface of the molybdenum electrode upon electrochemical oxidation, which increases the surface areas for biofilm adhesion and enhances the mass transfer of electrons [72]. Meanwhile, molybdenum exhibits biocompatible properties that enhance biofilm formation and support active microbial propagation, increasing microbial activity and generating electrons. Indeed, molybdenum is chemically stable and resistant to corrosion in many physiological conditions, which does not interfere with the cellular function of microbial cells, promoting cell viability and electrical stimulation when applied as electrode material [172]. Similarly, Shirkosh et al. [102] used a zinc electrode in a µMFC system cultured with *S. oneidensis* and reported a current density of 138,181 mA/m^2^ at a cell voltage of −0.3 V using a glucose medium. Zinc electrodes exhibit a higher OCP and current density than most carbonaceous-based electrodes in µMFCs inoculated with *S. oneidensis*, which could be ascribed to zinc material possessing a faster ion transfer rate in the electrolyte than the carbonaceous-based electrode and a higher reduction standard potential. Further, zinc-based µMFC could enhance the overall power performance by performing two series of redox reactions, including (1) redox reaction associated with zinc oxidation, which releases zinc ions into the anolyte as a new electrolyte; (2) redox oxidation associated with anolyte oxidation by electroactive bacteria [102].

However, the life span of metal-based µMFCs is constrained by the corrosive nature of the metal materials. In contrast, stainless-steel and surface-modified metal materials have gained increasing attention for use as an electrode material for µMFCs [173]. Stainless-steel exhibits passivation behavior, which will form a thin oxide layer on the surface when exposed to oxygen to resist corrosion and protect the underlying metal from further degradation. Moreover, stainless-steel possesses good electrical conductivity, allowing for efficient electron transfer between the electrode surface and the electrolyte. This conductivity facilitates electrochemical reactions at the electrode–electrolyte interface [94]. Liang et al. [174] proposed a carbon-coated stainless-steel electrode by inoculating *Geobacter* spp. in MFC. The electrochemical measurement of the modified electrode exhibited the highest current density of 13 A/m^2^, which is higher than the MFC conducted with an unmodified stainless-steel electrode (3 A/m^2^) through enhancing bioadhesive and electron transport rate. It has also been shown that flame-oxidized wolfram is a more effective anode material, and a facile wolfram modification yielded a power density of up to 1036 mW/m^2^ at ambient temperature [72]. A carbon nanofibers-PDMS coated stainless steel (CNF-PDMS/SS) electrode has been applied successfully in MFC to generate a maximum power density of 19 mW/m^2^ after 16 days of operation. Besides achieving higher power density output than unmodified electrodes, CNF-PDMS coating can enhance the corrosion resistance of stainless steel by reducing its corrosion rate from 367 µm/yr to 31 µm/yr [173]. Moreover, nickel nanostructure modification was performed to enhance anodic biofilm formation on the nickel foil electrode. Designing the µMFC using nickel nanostructure led to a higher volumetric power density of 343 W/m^3^ with an additional enhancement of 67% in the surface power density compared to the unmodified nickel electrode. The increased surface area due to the employment of nickel nanostructures on the anode side could increase the density of attached electroactive bacteria to the anode surface by enhancement of carbon source accumulation, thereby elevating the anolyte valorization and improving the mass transfer [153].

Aside from the electrode material selection, the diffusion layer existing over the electrodes also substantially influences the fuel cell output. Increasing the electrode surface area while assuming a homogenous diffusion layer could increase the overall power performance. Nevertheless, increasing the surface area of a single electrode pair µMFC system simultaneously increases the thickness of the diffusion layer, attributing to electron mass transfer limitation and a decrease in power density [175]. Kim and Chang [176] resolved this concern by investigating the effect of the electrode diffusion layer on MFC power generation. Using an immobilized inoculum source collected from a sludge tank in the Gwangju sewage treatment plant on the anode as bioanode, they revealed that splitting a single platinum-coated carbon cloth bioanode into nine simple bioanode while retaining the electroactive area and distributing them by some fixed distance fostered an increase in power output by a factor of 30%. In conclusion, the use of multiple smaller bioanodes and increased distance between the bioanodes could enhance the bioadhesive rate of electroactive bacteria while preventing an increase in the thickness of the diffusion layers [176]. A similar report was also put forward by Li et al. [177] wherein a multi-layer porous electrode consisting of five single-layer porous carbon paper electrodes was implemented in the vanadium µMFC system for bioelectricity production through continuous electrolyte replenishment. The system displayed a 1294% increment in power output with 120% lower internal resistance under a multi-layer flow-through porous electrode configuration. This could be attributed to the use of a multi-layer electrode which offers an uniform reaction rate and diffusion layers distribution in the porous electrode to cope with operating conditions resulting in a superior cell performance [177].

**Figure 13.**
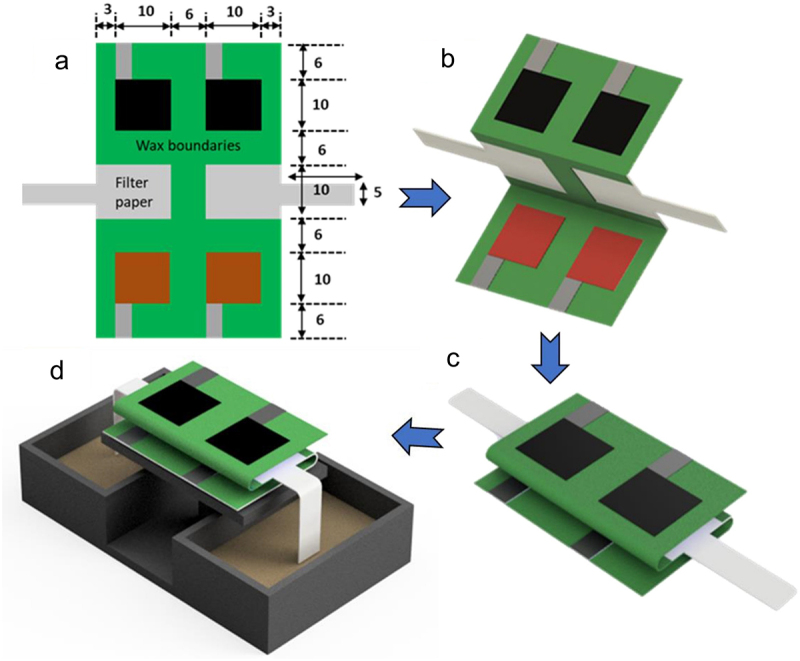
Schematic illustration and fabrication process for the origami array-type µMFC. (a) Schematic representation with dimension (mm), (b), (c) folded structure in 3D view, and (d) overview of origami array-type µMFC realization on 3D printed platform [178].

In another study, Jiang et al. [111] integrated six flow-through µMFCs in an array into a sandwich of two acrylic sheets. Operating the µMFCs, potassium ferricyanide and *S. oneidensis* MR-1 culture medium driven independently into catholyte/anolyte, the authors revealed 56 µA of total current with a 16.4-fold increase in volumetric density at 765 µA/cm^3^ and 4.3-fold decrease in current generation response time to achieve 80% of the peak output current than a single µMFC system [111]. A novel origami array-type µMFC for bioelectricity generation was developed by Rewatkar et al. [178] with a horizontally arrayed 1 cm^2^ manganese oxide nanoparticles coated carbon anode and 1 cm^2^ carbon cathode (Figure 13). The µMFC yielded a maximum power density of 15.9 µW/cm^2^ and OCP of 0.53 V. Further electrochemical analysis revealed that this integrated flow-through µMFC configuration improved interfacial nutrient assimilation to the bacterial colonization, biocompatibility, and direct electron transfer rate of the system.

## 5. Circular biorefinery approach- Microfluidic MFC/bioplastic closing the loop

**Figure 14.**
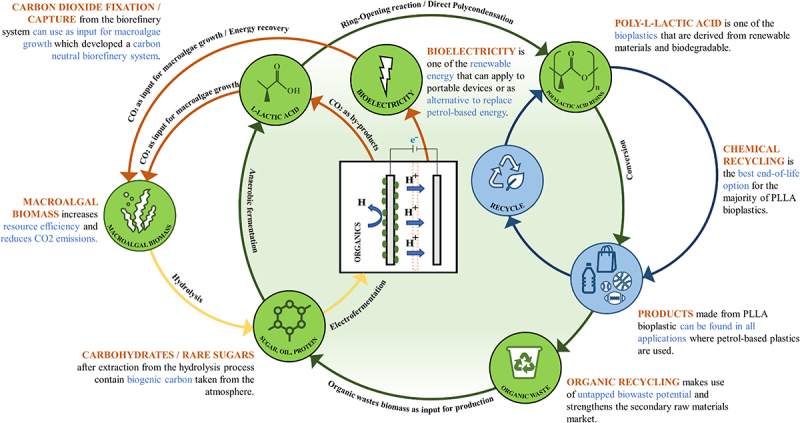
Schematic diagram of circular biorefinery and bioeconomy of microfluidic microbial fuel cell for bioelectricity and bioplastics production.

The circular biorefinery model has been employed to enhance the reuse and recycling of current biomass-derived wastes to produce new bioenergy and biochemicals using environmentally friendly techniques. Such the ‘take-make-recycle’ circular model offered a beneficial way to maintain continuous growth of the quality of life and economy by controlling the valorization of finite resources [179]. µMFCs can be promising circular biorefinery models as they can operate for biochemical production and energy recovery by valorizing various biogenic materials and wastes [154]. In this context, abundance macroalgae are a promising feedstock for developing the circular biorefinery model. The microbial reactions involved in the macroalgae hydrolyzate acting as a substrate in µMFC generate electrons and protons which can later be used for the co-production of bioelectricity and L-LA along with CO_2_ sequestration from organic matters present (Figure 14). Although CO_2_ is released into the anodic chamber during microbial reactions, that can be reused in the looping system for macroalgae re-growth and as a buffer medium for the system, making the µMFC a carbon-neutral technology. Compared to the conventional biorefinery model, µMFC consumes significantly lower energy rather than generating bioelectricity with EF reactions [70].

It is crucial to recognize that the macroalgal biomass upstream bioprocessing may significantly govern the range of downstream intended bioproducts. Moreover, depending on the targeted bioproduct of interest, selecting the most flawless approach or combination of approaches is paramount [10]. The interconnected structures of macroalgal cell walls are structurally and chemically more heterogenous than lignocellulosic materials as they are a polyphyletic group, which can be deciphered via the dynamic chemical diversity that presences between *Rhodophyta*, *Phaeophyta*, and *Chlorophyta*. Generally, macroalgal cell walls typically consist of a fibrillar skeleton material, known as cellulose and an amorphous enmeshed phycocolloids (carrageenan, agar, ulvan, glucuronan, or alginate) unique to each taxa to form a double fibrillar layer, which ultimately requires an effective hydrolysis assay to gain access to the internal cell membrane. This involves either conventional (chemo-catalytic and bio-catalytic) or innovative hydrolysis assay which disrupts the macroalgal cell wall constituents, followed by hydrolysis of polysaccharides into rare sugars [11]. The use of chemo-catalytic hydrolysis involving sulfuric acid (H_2_SO_4_) was explored by Hessami et al. [180] to enhance the extricated rare sugar (glucose and galactose) yield to 39.42% from red macroalgae *Gelidium elegans*.

From the economic standpoint of the macroalgal biorefinery, phycocolloids have frequently been the primary target for recovery due to being commercially valued [181]. Park et al. [182] and Alfonsín et al. [33] investigated the extraction of alginate and carrageenan, respectively, and assessed the appropriateness of the remaining residues as feedstock for rare sugar extraction using chemo-catalytic hydrolysis assay. Park et al. [182] identified that 40% of alginate can be extracted from *Laminaria japonica*, and 107.5 g/L of glucose was successfully extricated post hydrochloric acid hydrolysis. Similarly, Alfonsín et al. [33] suggested that a biorefinery approach using *Eucheuma denticulatum* for the production of carrageenan and rare sugars could be commercially viable and yielded 19.7% of extricated rare sugars corresponding to 0.30 g/L of glucose from the carrageenan-free residue through acid hydrolysis. Notably, unfavorable chemo-catalytic hydrolysis conditions could result in undesirable by-products including 5-hydroxymethylfurfural (HMF) and formic acid via degradation of extricated rare sugars, which is a side reaction of chemo-catalytic hydrolysis that is unable to surpass completely. These by-products will diminish the fermentation efficiency to produce the carbon-based end-point products, such as L-LA by hindering the protein synthesis and damaging the DNA of the fermentative bacteria [183].

As an alternative to chemo-catalytic hydrolysis, bio-catalytic, known as enzymatic hydrolysis with the utilization of enzymes to expedite the cleavage of macroalgal cell wall constituents and polysaccharides is preferred as this assay is environmentally benign [184]. The use of cellulase isolated from the halophilic bacterium *Vibrio parapaemolyticus* was explored by Hebbale et al. [185] to enhance rare sugars (glucose and rhamnose) extrication from green macroalgae *Enteromorpha intestinalis*. The authors revealed that enzymatic hydrolysis using cellulase from *V. parapaemolyticus* alone was sufficient to complete the saccharification of *E. intestinalis* with rare sugar yields of 90.59% and no 5-HMF formation. This is mainly due to cellulose isolated from *V. parapaemolyticus* is composed of thermostable and acidic endoglucanase, which shows higher catalytic efficiency on acid-sensitive intermolecular glycosidic bonds cleavages compared to basic endoglucanase [185]. In fact, the cellulase from *V. parapaemolyticus* contains the lysine decarboxylase (cadA) genes that will degrade the lysine content in the macroalgal biomass to maintain the optimum pH conditions (pH 3–8) of the system, thereby extricated rare sugars yield can be enhanced simultaneously by aggrandizing the enzyme activity [186]. Further, Rocher et al. [24] performed the enzymatic hydrolysis of brown macroalgae *Ecklonia maxima* using yeast *Saccharomyces cerevisiae* and justified that the yield of extricated glucose could be significantly improved from 29% to 66% by genetically modified yeast with laminarinase-encoding genes from *Trichoderma viride* and *Rasamsonia emersonii*. This is attributed to the *S. cerevisiae* cannot produce sufficient of its native laminarinase-like enzyme for effective hydrolysis of laminarin [24]. Although high rare sugar yields can be observed, this assay is constrained by the extended retention times of hydrolysis ranging between 1 and 5 days and high production costs [24,185]. Thus, the use of enzymatic hydrolysis usually implies with pretreatment approach to reduce the retention times of the process which is indicated as a two-step hydrolysis assay and considered not viable from the economic perspective [184].

To improve the setbacks of conventional hydrolysis assays, eco-friendly and innovative hydrolysis assays are increasingly developed, including microwave-assisted hydrolysis and ozonolysis [12,187]. Microwave-assisted acid hydrolysis has been employed successfully to extract galactose from *E. denticulatum* carrageenan under 120°C with an operation duration of 25 min [12]. The authors concluded that 50.7% of the galactose recovery rate, corresponding to 27.9 ± 1.69 g/L of galactose can be achieved along with a low 5-HMF of 0.74 ± 0.18 g/L from the *E. denticulatum* carrageenan with the involvement of 230 W microwave power [12]. Cao et al. [188] further applied higher microwave power (1900 W) to enhance the acid hydrolysis of *Gracilaria lemaneiformis* under the optimized temperature of 180°C with 0.2 M H_2_SO_4_, and the extricated rare sugars (galactose and glucose) yield reached up to 73.3% with a retention time of 20 min, which is sixfold lesser compared conventional acid hydrolysis assay. They revealed that the superficial heat transfer environment offered by microwave irradiation to the macroalgae biomass can improve the extricated rare sugars yield and retention time of the process whilst simultaneously limiting the formation of 5-HMF [188]. The use of microwave irradiation reduced the retention time and by-products formation, which paths a new route for the hydrolysis of macroalgal biomass for the circular biorefinery; however, bottlenecks associated with the high-power consumption for microwave heating at the targeted temperature on a commercial scale were raised.

In contrast, the ozonolysis assay has been widely applied as a lignocellulosic pretreatment process for delignification. However, it has not been extensively explored as a hydrolysis assay for macroalgal biomass as they are less recalcitrant due to a lack of lignin complexes [189]. The ozonolysis assay utilizes ozone (O_3_) to treat the biomass cell walls and selective carbohydrate degradation at ambient temperature and pressure with minimal effects on rare sugars, leading to lower yield in 5-HMF generation in the extricated rare sugars tenable for fermentation. As an electrophilic and triatomic molecule, O_3_ is highly reactive toward the electron-rich cell wall matrix and carbohydrates due to an electron deficiency in terminal oxygen during resonance [190]. The reactions of O_3_ with carbohydrate molecules involve an initial electrophilic attack, resulting in the formation of carboxyl and carbonyl groups followed by hydroxylation of the carbonyl groups, which increases electrophilic substitution reactivity of the compounds for glycosidic bond cleavage [191]. Although O_3_ is an efficient oxidizing agent (E^0^ = 2.07 V, 25°C), O_3_ alone is relatively ineffective in oxidizing cellulose surfaces [189]. Thus, Tamilarasan et al. [187] explored hydrothermal ozonolysis to improve rare sugar recoveries from green macroalgae *Chaetomorpha antennina*. Compared with the control case (ozonolysis alone), total rare sugar recovery rates and energy ratio increased 2.1 and 2.4 times with water as a hydrolysis solvent. This attributed to treatment with O_3_ in an aqueous medium that generates a reactive radical known as hydroxyl through the formation of superoxide, which reacts with the cell wall matrix resulting in random cleavage of hydrogen bonds to extricate carbohydrate contents. In addition, soluble low molecular weight compounds, mainly organic acids including acetic acid and formic acid, will be released during ozonation, resulting in a decrease in the acidification of the solution that can expedite the cleavage of acid-sensitive glycosidic bonds [187]. Further, Sulfahri et al. [192] significantly improved total rare sugar extricate yield in red macroalgae *Kappaphycus alvarezii* from 0.16 g/g to 0.52 g/g with ozone-assisted enzymatic hydrolysis. Hence, ozone-assisted hydrolysis assay is preferred for rare sugar extrication in terms of high hydrolytic efficacy and low energy consumption, which allies with the criteria embedded within the circular biorefinery concept.

Anaerobic fermentation (AF) is the indispensable sequential stage in the circular biorefinery loop for the valorization of extricated rare sugars or glycolytic intermediates toward L-LA production using acid-tolerant LAB. Among thousands of strains of identified LAB, *Bacillus spp*. and *Lactobacillus spp*. are the prominent microbial that have been perceived as the most crucial contributing to beneficial effects in L-LA fermentation using carbon-rich rare sugars as substrate. A typical superiority of these LAB strains for L-LA production is offering strength to growth under high temperatures up to 50°C, utmost resistance to contamination, and less stringent nutritional requirements [193]. Wu et al. [194] concluded that 7.02 g/L (48.48%) of L-LA can be attained during the fermentation of acid-modified *Ulva lactuca* hydrolyzates using 10% (v/v) *Lactobacillus plantarum* under batch mode at 37°C for 24 h. Under the same inoculum density, the fermentation process for acid-modified *E. denticulatum* cellulosic residues was optimized to achieve a L-LA yield of 98.6%, corresponding to 14.02 g/L of L-LA (Chai et al., 2021). Lin et al. [47] also explored the mixed microbial culture of combined *L. acidophilus* and *L. plantarum* strains for L-LA fermentation from red macroalgae *Gracilaria sp*. hydrolyzates, finding remarkable L-LA titer enhancements by merely 30% with L-LA yield of 64.72% compared to monoculture. This may be due to the probiotic bacterium *L. acidophilus* will excrete proteinaceous bacteriocins including lactocin B, acidocin A, and acidocin B during the L-LA fermentation as nitrogen sources to stimulate *L. plantarum* strain growth [195]. Tong et al. [12] further enhanced the L-LA titer by employing various inoculum densities of *Bacillus coagulans* at 37°C for 14 h and revealed that the L-LA conversion yield was improved from 19.93 to 22.49 g/L with an increment of 88.62% when 4% (v/v) *B. coagulans* was applied compared to only 2% (v/v). In conclusion, a higher volumetric productivity of L-LA and a shorter metabolization rate of LAB can be achieved by employing a high inoculum density culture as it offers a rapid fermentation process between LAB and rare sugars [12].

Apart from the generation of intended bioproducts, the successful reduction of waste within a biorefinery process is a covetable goal that ought to be achieved by adopting green technologies to incorporate within the cascading biorefineries. Lin et al. [118] accomplished this during their research which showed the viability of generating bioelectricity from residues of glucose and glycolytic intermediates that remained post L-LA production. The study demonstrated this integrated biorefinery assay of L-LA and bioelectricity production in a MFC system using a combined bacterium strain of *S. oneidensis* and engineered *S. cerevisiae* for the EF process. L-LA yield of 7.50 g/L was attained from the glucose medium, after which the residues were utilized as substrates in MFC to generate bioelectricity. The output voltage and power density obtained from this MFC system were 355 mV and 123.41 mW/m^2^, respectively [118]. Tejedor-Sanz et al. [97] concluded that MFC is a sustainable CO_2_ sequestration platform, where the electroactive bacterium *Lactiplantibacillus plantarum* can convert CO_2_ released during L-LA fermentation into a variety of value-added bioproducts (acetic acid, ethanol, and bioelectricity). Further, Shirkosh et al. [102] enhanced the bioelectricity generation by downscaling the MFC into microfluidic environments, finding a markedly technological advantage by generating higher power density output 35,294 mW/m^2^ compared to MFC. These findings revealed that µMFC is an emerging waste-to-chemical/energy platform for CO_2_ fixation and multiple carbon-end point bioproduct generation from a single substrate, indicating a significant opportunity for developing a circular biorefinery model.

In the circular biorefinery model, the L-LA produced will be further processed into biodegradable PLLA polymer for bioplastic application via a Ring-Opening lactide reaction or a Direct Poly-Condensation of L-LA [41]. The end-of-life for such bioplastics can be recycled or biodegraded using microorganisms. PLLA-based products can be subjected to chemical recycling. In detail, chemical recycling is a depolymerization process using mild acids or solvents. Then, the depolymerized components can be repolymerized to form valuable products [196]. In addition, Adhikari et al. [197] studied the biodegradation of PLLA-based products. The authors reported that the fragments components of the PLLA polymers via biodegradation can be utilized as substrates for either anaerobic fermentation or EF to exploit the untapped potential [197]. The energy recovery from the biodegradation process can be used further for the production of power or heat [198]. Thus, a close-loop circular biorefinery model is demonstrated.

## 6. SWOT analysis of microfluidic microbial fuel cell toward bioelectricity and bioplastic production for circular biorefinery

Technology Transfer is essential to move technology from research labs toward commercialization. Thus, the strengths, weaknesses, opportunities, and threats (SWOT) analysis of a raised technology product must be conducted to identify its future prospects. An appropriate SWOT analysis would be beneficial to transfer the obtained scientific findings to industrial- or pilot-scale applied technology. In the following sections, the possibility for the transfer of µMFC technology to fruition will be analyzed regarding the strengths of this technology, technological limitations, challenges, and future prospects.

### 6.1. Main strengths and weaknesses of microfluidic microbial fuel cell

The most vital strength point of µMFC is the possible use of organic substrate or wastewater as a fuel source to initiate biocatalytic environmental remediation and co-production of biochemicals and bioelectricity. Hence, this unique property provides a sustainable approach for the processing of multiphase waste treatment and biomass utilization while reducing CO_2_ emissions, which is accorded with the UN Sustainable Development Goals [175,199]. On account of its modest size, µMFC can be easily scaled up or down, offering versatile applications, including bioenergy production, wastewater remediation, biosensors, and remote power generation, and enabling integration into lab-on-chips devices [147]. Further, the compact nature of microchannels in µMFC minimizes diffusion limitations by increasing the surface-to-volume ratio of the system, which promotes mass transfer capabilities regarding electron and nutrient transfer between the microbes and electrodes. This allows for improved accessibility of microbes to the substrates, enhancing the reaction kinetics and power generation [153]. The elimination of PEM in the µMFCs is an economically sustainable strategy that simultaneously reduces the internal resistance attributed to proton transport, improving the efficiency of the µMFCs. Moreover, µMFCs possess the ability for multiplexing and parallelization, allowing simultaneous operation of multiple microchannels or units, which enables higher throughput and increased power output [152].

Because of the precise microscale features, the fabrication of µMFCs can be technically challenging and require specialized equipment and materials. Achieving reliable and reproducible fabrication processes for µMFCs can be time-consuming and costly. However, despite having high mass transfer capabilities, µMFCs are still hurdled by mass transfer limitations. The limited volume microchannels in µMFC can overload the substrate [147]. Besides, µMFC may require integration with external components, including pumps, monitoring systems, and sensor, which contribute to the complexity and challenges in terms of system reliability, compatibility, and overall performance. Additionally, establishing long-term operational stability and performance consistency of µMFs can be challenging. This is attributed to the performance of µMFCs relying on forming and maintaining biofilms on the electrode surface. The intricate fluidic conditions, limited space, and potential for biofilm degradation are the several factors that made biofilm control a difficult process [148]. Lastly, scaling up µMFCs from lab-scale to practical applications while retaining performance and cost-effectiveness is limited due to mass production and system integration [167].

### 6.2. Challenges and future prospects

As conventional MFCs are a well-established technology and have been extensively studied with a mature research base and developed protocols, this could limit the public perception and acceptance of µMFCs. Furthermore, the involvement of intricate designs, specialized fabrication techniques, and complex fluidic dynamics in the study of µMFCs raises challenges regarding operation and maintenance, which may also hinder their widespread adoption [200]. Besides, recent studies on µMFC have been made without fully employing biofilm engineering and fabrication techniques that are specifically designed to enhance µMFC performance significantly. Consequently, the development of 3D printing technology for anodes with biofilm engineering has enormous potential for supporting the development of high-performance µMFCs in the future, as they possess the ability to enhance EET to stimulate power generation by several orders of magnitude [160]. Immobilization of exoelectrogens in the mesh-like electrodes can also be considered a potential advancement to control and maintain biofilm growth. Regarding microscale features, µMFC offers opportunities to develop miniaturized bioenergy devices suitable for portable and on-site power generation in resource-limited or remote settings. Then, the study of µMFC can be integrated with lab-on-chips systems, allowing for simultaneous energy generation and biosensing, enabling multifunctional devices. To expand the market opportunities and adoptions, researchers should tailor µMFC design for specific applications such as biomedical devices or environmental monitoring systems, exposing the usage of µMFCs. Moreover, µMFC competitiveness can be further increased by understanding the thermodynamics of EET at the microscale and maximizing the generation of all available high-value components from the single substrate through cascading biorefinery. The abovementioned prospects guide future work toward a full understanding of the high performance of µMFC system development as a viable technology.

## 7. Conclusions

This review delineated the in-built potential of macroalgae in terms of bioproducts recovery and simultaneously diminishing the carbon footprint with a sustainable perspective. There are several pathways for the current sustainable energy transition. MFC technologies provide a potential approach for cascading biorefinery to exploit the co-production of bioelectricity and biochemicals from the organic substrate that could replace conventional sources of petrochemicals. Bioelectricity and L-LA generated from the metabolism of exoelectrogens or LAB can be maintained to provide a reliable continuous source of renewable energy and bioplastic production. Nevertheless, the commercialization of MFC is a hurdle associated with insufficient power generation. Currently, investments in circular biorefinery are focused on using µMFC system to enhance the power output and achieve a better conversion yield of L-LA. It promises to be the most potential biorefinery model to address the futuristic circular biorefinery with more innovation in the near future. Appropriate economic, technical, and scientific strategies in a multi-disciplinary approach can lead researchers to pursue a sustainable algae-to-chemical/energy system by addressing the circular biorefinery goals and bridging the research gaps between waste remediation and product recovery.

## Data Availability

The data that support the findings of this study are available from tan.s@curtin.edu.my.
